# Attenuated *Salmonella* Typhimurium Lacking the Pathogenicity Island-2 Type 3 Secretion System Grow to High Bacterial Numbers inside Phagocytes in Mice

**DOI:** 10.1371/journal.ppat.1003070

**Published:** 2012-12-06

**Authors:** Andrew J. Grant, Fiona J. E. Morgan, Trevelyan J. McKinley, Gemma L. Foster, Duncan J. Maskell, Pietro Mastroeni

**Affiliations:** Department of Veterinary Medicine, University of Cambridge, Cambridge, United Kingdom; Stanford University School of Medicine, United States of America

## Abstract

Intracellular replication within specialized vacuoles and cell-to-cell spread in the tissue are essential for the virulence of *Salmonella enterica*. By observing infection dynamics at the single-cell level *in vivo*, we have discovered that the *Salmonella* pathogenicity island 2 (SPI-2) type 3 secretory system (T3SS) is dispensable for growth to high intracellular densities. This challenges the concept that intracellular replication absolutely requires proteins delivered by SPI-2 T3SS, which has been derived largely by inference from *in vitro* cell experiments and from unrefined measurement of net growth in mouse organs. Furthermore, we infer from our data that the SPI-2 T3SS mediates exit from infected cells, with consequent formation of new infection foci resulting in bacterial spread in the tissues. This suggests a new role for SPI-2 *in vivo* as a mediator of bacterial spread in the body. In addition, we demonstrate that very similar net growth rates of attenuated salmonellae in organs can be derived from very different underlying intracellular growth dynamics.

## Author Summary

High quality science has been published concerning the dynamics of infectious disease spread through communities of people or animals, but less work has been done to understand infectious disease dynamics within the host. Many conclusions about how infectious agents work are based on experiments in isolated monocultures of cells or in somewhat crude experiments in whole animals. Understanding this complex process in whole animals is the next major challenge for infectious disease biologists, and is required if intervention strategies to prevent and cure infectious diseases are to be improved and targeted effectively. Bacteria of the species *Salmonella enterica* are a threat to public health, causing a wide range of life-threatening diseases in humans and animals world-wide. *In vitro* cell experiments and inference from measuring net growth kinetics in mouse organs suggest that intracellular replication of *S. enterica* requires proteins delivered by the *Salmonella* pathogenicity island 2 (SPI-2) type 3 secretion system (T3SS) and that mutants in SPI-2 cannot replicate efficiently intracellularly. However, by observing directly infection dynamics at the single-cell level *in vivo*, we show that SPI-2 T3SS mutants can replicate to high intracellular densities in phagocytes in the organs of infected animals, but appear unable to leave infected cells.

## Introduction

Infection of a host by a bacterium is a dynamic process that can be measured and analyzed at different scales. Many experimental systems for understanding infection measure the overall increase or decrease in numbers of bacteria in different organs in the host over time. Often, fine-structure dynamics of the interactions between bacteria and host cells are measured using *ex vivo* or *in vitro* systems, and these results are used to infer mechanisms that explain net patterns of survival *in vivo*. Most of these inferences have not been tested experimentally in whole-animal models of infection. It is clear that similar overall net survival patterns in organs might be derived from very different underlying processes. For example, an apparent net lack of growth could arise when a bacterium grows very slowly or not at all in an organ over a period of time, or it could arise from a bacterium replicating quickly but also being killed at a similar rate, giving the appearance of a net lack of growth. Unraveling these complex underlying infection dynamics is important for a full understanding of the host-pathogen interaction, and is crucially important if intervention and prevention strategies are to be improved and applied to maximum effect.

An ideal model system with which to study these fundamental dynamic mechanisms *in vivo* is provided by invasive *Salmonella enterica* serovar Typhimurium infections of mice. In this system the bacteria live within spleen and liver phagocytes [1] and replicate inside a specialized, membrane-bound vacuole: the *Salmonella*-containing vacuole (SCV). Wild-type salmonellae in susceptible mice grow rapidly in the organs, at a net rate approximating to a ten-fold increase per day. On the other hand, live attenuated vaccine strains containing mutations in defined genes show extremely slow or no net growth, and then are cleared from the organs, in the process stimulating protective immunity. Of particular interest here are those vaccine strains that are mutated in the same genes as new vaccine candidates being tested in the field for protection of humans against typhoid fever caused by *S*. Typhi. Prime examples are mutants that lack the type 3 secretion system (T3SS) encoded by *Salmonella* Pathogenicity Island 2 (SPI-2) [2].

In *S. enterica*, as in other bacterial species, T3SSs have evolved to deliver proteins from the bacterium into the host cell [3]. SPI-2 T3SS is required for replication of *S*. Typhimurium in some cell lines *in vitro* [4]–[7], and a reasonable assumption from this is that SPI-2 is also required for intracellular replication of salmonellae in the host animal [5], [6], [8]. This idea is supported by the fact that mutants lacking SPI-2 survive in the livers and spleens of infected animals, but show limited or no net growth in numbers [5], [8], [9].

Previously, we have used multi-color fluorescence microscopy (MCFM) to image host cells in histological sections of infected mouse organs, counting the number of bacteria per cell and the distribution of infected cells throughout infected organs. This is a direct way of observing fine-structure infection dynamics cell-by-cell, and it reveals the intracellular bacterial growth dynamics that underpin the net dynamics observable at the whole organ level. We found that when wild-type salmonellae are replicating rapidly in the organs, the number of bacteria per infected phagocyte is unexpectedly low and remains low [10]–[13]. Consequently, as the infection progresses and viable bacterial numbers per organ increase, the bacteria must undergo only a few replication cycles before they escape from the originally infected cells and disperse to infect new cells, where new infection foci emerge and the cycle repeats itself [10]–[15].

We used this more sophisticated understanding of the dynamics of growth of salmonellae in murine organs to address the hypothesis that SPI-2 is required for intracellular growth of the bacteria *in vivo*. Based on our previous studies, we predicted that a mutant lacking the SPI-2 T3SS would be present in the organs at the very low intracellular densities typical of slowly dividing strains [10], [11]. However, we show that SPI-2 T3SS mutants can replicate to high intracellular densities in the spleens and livers of infected animals, but appear less able to leave infected cells than wild-type salmonellae, restricting bacterial dispersal through the tissues and dramatically reducing the formation of new foci of infection in the tissues.

## Results

### SPI-2 T3SS mutants are present at higher densities per cell on average than the wild-type

**Figure 1:**
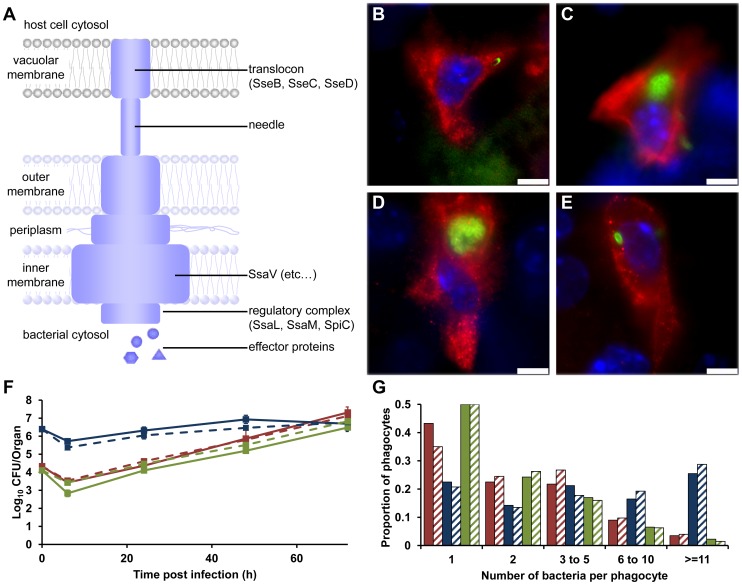
SPI-2 T3SS mutants can grow to high intracellular densities in CD18^+^ cells *in vivo*. (A) Model of the SPI-2 T3SS. SseB forms part of the translocon; SsaV is located in the inner membrane. (B–E) Representative fluorescence micrographs of *Salmonella* within phagocytes in infected livers of C57BL/6 mice at 72 h p.i. (B) S12023; (C) S12023 *ssaV*; (D) S12023 *sseB*; (E) S12023 *sseB*(psseB) [CD18^+^ cells (red), *Salmonella* (green), nucleic acid is stained with DAPI (blue), scale bars, 5 µm]. (F and G) C57BL/6 mice were infected i.v. with ∼Log_10_ 4.3 CFU ( = 2.15×10^4^ CFU) of S12023, ∼Log_10_ 6.4 CFU ( = 2.45×10^6^ CFU) of S12023 *sseB*, or ∼Log_10_ 4.1 CFU ( = 1.27×10^4^ CFU) of S12023 *sseB*(psseB). (F) Net bacterial numbers in livers (unbroken lines) and spleens (dotted lines) were determined between 6 to 72 h p.i. from 4 mice per strain per time point (results are expressed as mean Log_10_ viable count ± standard deviation). (G) The proportion of infected phagocytes relative to the number of bacteria contained within each phagocyte is shown for livers (solid bars) and spleens (diagonal shading) at 72 h p.i. based on the counts obtained from 400 infected phagocytes per strain, from tissue obtained from 4 mice per strain [S12023 - red; S12023 *sseB* - blue; S12023 *sseB*(psseB) – green].

Initially we studied a mutant of *S*. Typhimurium strain NCTC 12023 (S12023) lacking the *ssaV* gene [16], which is unable to assemble the SPI-2 secretory machinery [17] (Figure 1A). The overall net growth of this mutant in infected organs is dramatically reduced compared with the wild-type parent, as determined by colony counts on organ homogenates [6], [7]. The wild-type exhibited the expected infection dynamics at the cellular level, with low numbers of bacteria per cell at 72 h post infection (p.i.) (Figure 1B), despite rapid net growth per organ, this being entirely consistent with our previous findings [10]–[13]. On the other hand, most unexpectedly, at 72 h p.i. the *ssaV* mutant was present at high numbers of bacteria per cell (Figure 1C) and was much less dispersed throughout the tissue than the wild-type bacteria. This was very surprising. We expected it to be distributed mostly as one bacterium per cell.

To test whether this observation was generally true and not limited to the bacterial strain used, we repeated the analysis using *ssaV* mutants in two other strains (SL1344 and C5), and observed similar results, with, on average, high numbers of *ssaV* mutant bacteria per cell (data not shown).

To investigate whether these observations were the result of the *ssaV* mutation specifically, or whether this pattern was a general phenomenon, we studied a different SPI-2 mutant lacking *sseB* (SseB forms part of the SPI-2 T3SS translocon) (Figure 1A). S12023 *sseB* [5] and S12023 *ssaV* behaved similarly in terms of slow-to-negligible net growth per organ *in vivo*, apparently paradoxically high intracellular densities (Figure 1D), and relative lack of dispersal throughout the tissues. Complementation of *sseB* using plasmid psseB [5] resulted in the intracellular densities and intra-organ dispersal pattern returning to wild-type (Figure 1E).

To ensure that the SPI-2 T3SS mutants being observed were indeed intracellular and not simply aggregated around the outside of the cell, we used confocal microscopy, staining for markers for phagocyte cell membranes. Three-dimensional reconstruction of these images showed that the bacteria were completely enclosed within phagocyte membranes, and that they are therefore intracellular (data not shown). Thus mutants lacking SPI-2 T3SS can grow to high numbers in some infected cells *in vivo*, despite having a low net growth pattern per organ.

Given that these results were very unexpected, we proceeded with a deep and rigorous quantification of intracellular bacterial densities in the tissues of mice infected with wild-type S12023, S12023 *sseB*, or the complemented S12023 *sseB*(psseB). We therefore compared, at 72h p.i., the overall net levels of viable bacteria per liver or spleen (Figure 1F) with underlying bacterial loads within CD18^+^ phagocytes (Figure 1G). Because of the markedly different net growth rates per organ of the wild-type and mutant bacteria, different initial doses of bacteria were used to ensure that the total number of bacteria per organ per strain would be similar at 72 h p.i.. To assess formally the intracellular bacterial distributions between the strains, proportional odds ratios (ORs) and 95% credible intervals were generated from a Bayesian ordinal regression model (for more details see Supporting Information – Protocol S1). At 72 h p.i. the intracellular bacterial distributions of the wild-type bacteria and the complemented mutant were both as expected, based on our previous work [10]–[13], with intracellular densities heavily skewed towards low numbers (Figure 1G, Figure S1 and Table S1). Conversely, despite its low net growth rate per organ (Figure 1F), there is clear evidence that S12023 *sseB*-infected phagocytes are more likely to have higher intracellular bacterial densities than the wild-type or complemented strains (Figure 1G, Figure S1 and Table S1).

Our data support the hypothesis that SPI-2 T3SS mutant bacteria can multiply inside phagocytes. To account for the low overall net bacterial growth per organ, this replication may occur in fewer cells, may be slower or may initiate at wild-type rate and then slow down or stop as bacterial numbers per cell become high (the latter density-dependent scenario being predicted in our previous mathematical models [11]).

### SPI-2 T3SS mutants are present in far fewer infected foci per organ than the wild-type

**Figure 2:**
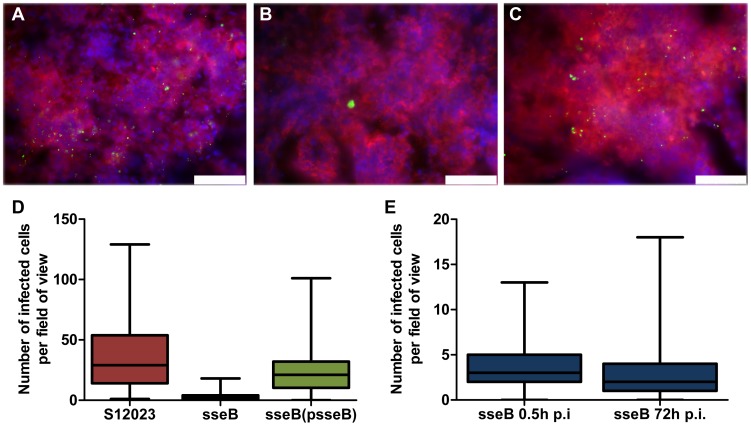
SPI-2 T3SS mutants are present in far fewer infected foci per organ than the wild-type. (A to C) Representative fluorescence micrographs of *Salmonella* within phagocytes in infected livers of C57BL/6 mice at 72 h p.i.. (A) S12023; (B) S12023 *sseB*; (C) S12023 *sseB*(psseB) [CD18^+^ cells (red), *Salmonella* (green), nucleic acid is indicated by DAPI (blue), scale bars, 75 µm]. (D and E) Box and whisker plots showing the median, interquartile range and maximum and minimum number of infected cells per field-of-view in spleens for (D) S12023, S12023 *sseB* and S12023 *sseB* p(*sseB*) at 72 h p.i., obtained from 100 random fields from 4 mice for S12023 and S12023 *sseB*(psseB) infected tissue and 200 random fields from 7 mice for S12023 *sseB* infected tissue, and (E) S12023 *sseB* at 0.5 and 72 h p.i., obtained from 100 random fields from 3 mice for S12023 *sseB* infected tissue at 0.5 h p.i., and 200 random fields from 7 mice for S12023 *sseB* infected tissue at 72 h p.i.

The higher intracellular densities combined with our initial observations that SPI-2 T3SS mutant bacteria are less dispersed in the tissues were suggestive of a role for SPI-2 T3SSs as a mediator of bacterial escape from infected cells, dispersion through infected tissues and increase in the number of infection foci. To investigate this, we quantified the number of infected cells per field-of-view throughout the tissues at 72 h p.i. (Figures 2A–D). The *sseB* mutant was substantially less dispersed throughout the tissue than the wild-type or complemented bacteria (Figures 2A–D, Figure S2 and Table S2). The wild-type bacteria and the complemented mutant had formed many more infection foci than the S12023 *sseB* mutant (Figures 2A–D, Figure S2 and Table S2) despite there being similar total bacterial numbers in the tissues at this time (Figure 1F). There was a small decrease in the number of infection foci between 0.5 h p.i. and 72 h p.i. in animals infected with the S12023 *sseB* mutant further confirming the impaired dispersion of this strain (Figure 2E, Figure S2 and Table S2).

As further corroboration of our data we generated a mutant lacking *spiC*, which is essential for SPI-2 T3SS protein secretion and effector translocation by interacting with SsaM and SsaL (Figure 1A), themselves encoded within the *spi-2* locus [18]–[21]. Some studies have proposed that SpiC may be exported by the SPI-2 T3SS into the host cell cytosol [22], where it interacts with host proteins, TassC [23] and Hook3 [24], which are implicated in cellular trafficking and the activation of signal transduction pathways [25]–[28]. We found that a mutant lacking *spiC* gave a similar phenotype to the *sseB* and *ssaV* mutants in terms of intracellular bacterial densities and tissue dispersion (Figure S3 and Table S2). In addition, we found that an *ssaM* mutant had similar intracellular bacterial densities to a *spiC* mutant (data not shown).

The SPI-2 T3SS mutants (*ssaV*, *sseB*, *spiC*, *ssaM*) are therefore present in far fewer infected cells per organ but at higher densities per cell than the wild-type.

### SPI-2 T3SS mutants seem unable to exit infected cells and form new infection foci *in vivo*

The accumulation of SPI-2 T3SS mutant bacteria inside cells is most likely explained by the intracellular replication of an infecting bacterium, but all other possible explanations had to be explored before this unexpected mechanism could be supported.

**Figure 3:**
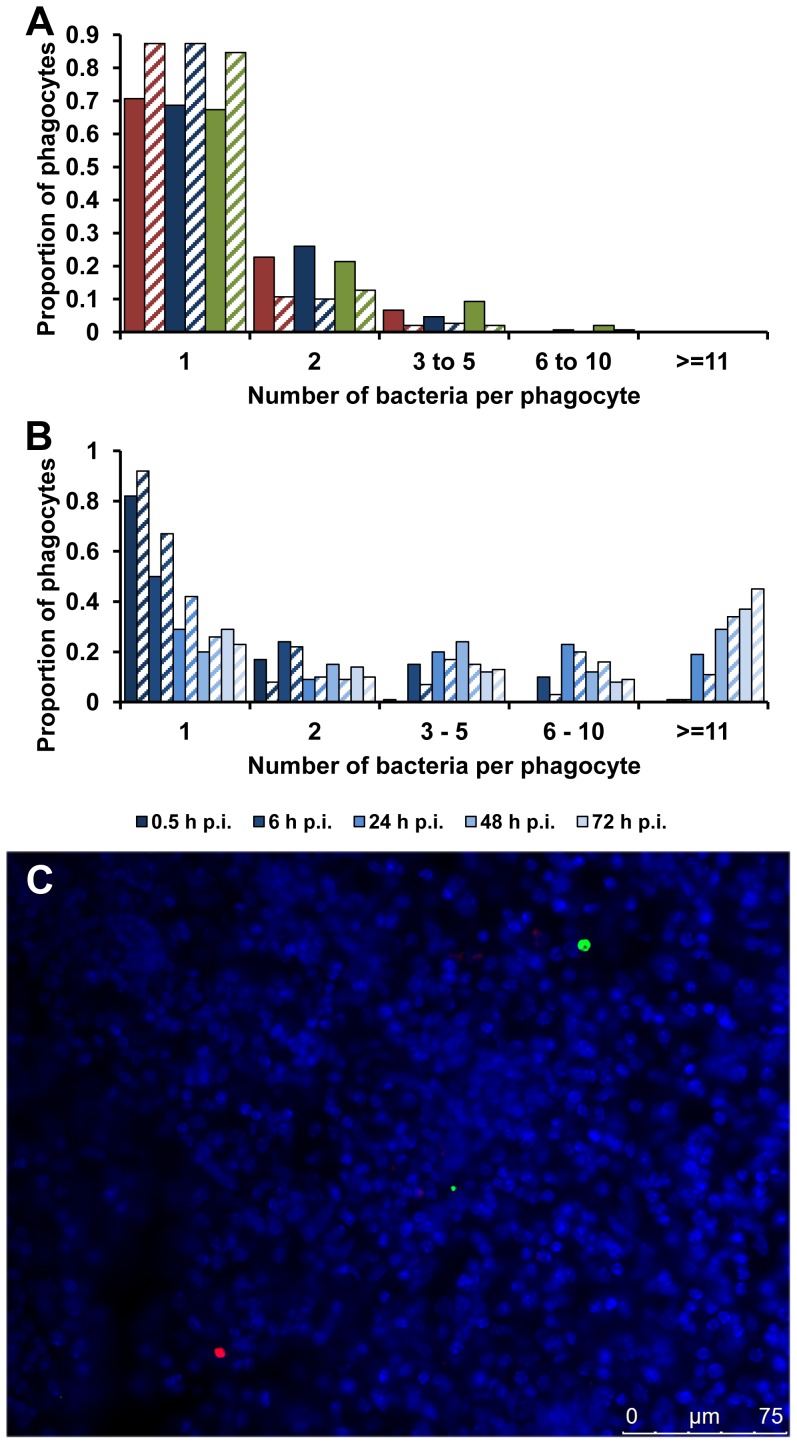
Accumulation of SPI-2 T3SS mutants inside cells is due to clonal expansion of a bacterium. (A) C57BL/6 mice were infected i.v. with ∼Log_10_ 6.4 CFU ( = 2.44×10^6^ CFU) of *Salmonella* S12023, ∼Log_10_ 6.5 CFU ( = 2.86×10^6^ CFU) of S12023 *sseB*, or ∼Log_10_ 6.3 CFU ( = 2.17×10^6^ CFU) of S12023 *sseB*(psseB). The proportion of infected phagocytes relative to the number of bacteria contained within each phagocyte is shown for livers (solid bars) and spleens (diagonal shading) at 0.5 h p.i. based on the counts obtained from 150 infected phagocytes per strain, from tissue obtained from 3 mice per strain [S12023 - red; S12023 *sseB* - blue; S12023 *sseB*(psseB) – green]. (B) C57BL/6 mice were infected i.v. with ∼Log_10_ 6.4 CFU ( = 2.64×10^6^ CFU) of S12023 *sseB*. The proportion of infected phagocytes relative to the numbers of bacteria contained within each phagocyte is shown for livers (solid bars) and spleens (diagonal shading) at different time points between 0.5 and 72 h p.i. inclusive, based on the counts obtained from 100 infected phagocytes per organ, per time point, from tissue obtained from 4 mice per time point. (C) C57BL/6 mice were infected i.v. with ∼Log_10_ 6.3 CFU ( = 1.99×10^6^ CFU) of SL5559 *sseB* and SL5560 *sseB* bacteria into the same animal *via* a single injection. Representative fluorescence micrograph of *Salmonella* SL5559 *sseB* and SL5560 *sseB* within phagocytes in an infected spleen at 72 h p.i. [SL5559 *sseB* (green), SL5560 *sseB* (red), nucleic acid is stained with DAPI (blue). Scale bar, 75 µm].

The first possibility tested was that the high intracellular densities seen with the SPI-2 T3SS mutants depended on phagocytes taking up clumps of bacteria. Groups of mice were infected with the same dose of the wild-type or the mutant bacteria. At 0.5 h p.i., when there is no evidence of any discernible bacterial growth or death [14], most of the bacteria within CD18^+^ phagocytes were present as a single bacterium per cell with negligible differences in the intracellular bacterial distributions between any of the strains (Figure 3A, Figure S1 and Table S1). This is consistent with individual resident macrophages taking up single bacterial cells from the blood and eliminates the clumping hypothesis.

We proceeded with a rigorous quantification of intracellular bacterial densities in the tissues of mice infected with S12023 *sseB*, and discovered that he proportion of host cells containing large numbers of intracellular bacteria increased with time during an infection (Figure 3B, Figure S4 and Table S1). In addition, the number of bacteria in the heavily infected cells increased with time, with some of the heavily infected cells containing ∼100 bacteria at 72 h p.i. (data not shown). This data is consistent with the bacteria growing inside the cell.

Alternatively, although extremely unlikely, high intracellular numbers could be reached by phagocytic cells moving around the organ and taking up bacteria on the way, a mechanism illustrated by analogy to the arcade game “Pac-Man”. To test the “Pac-Man” idea, we performed simultaneous infections where we inoculated two *Salmonella sseB* mutants (expressing different LPS O antigens enabling the mutants to be differentially visualized by immunostaining in tissue sections) into the same animal. During the course of the infection the two strains remained segregated to different phagocytes and infection foci (Figure 3C), indicating that the high numbers of bacteria in each cell are not the result of a “Pac-Man” mechanism.

The most likely explanation for the high intracellular numbers of SPI-2 T3SS mutants is that there is replication and clonal expansion of an individual bacterium, and that therefore SPI-2 is not an absolute requirement for intra-macrophage replication of salmonellae.

### Similar net growth kinetics can be the result of very different intracellular infection dynamics

**Figure 4:**
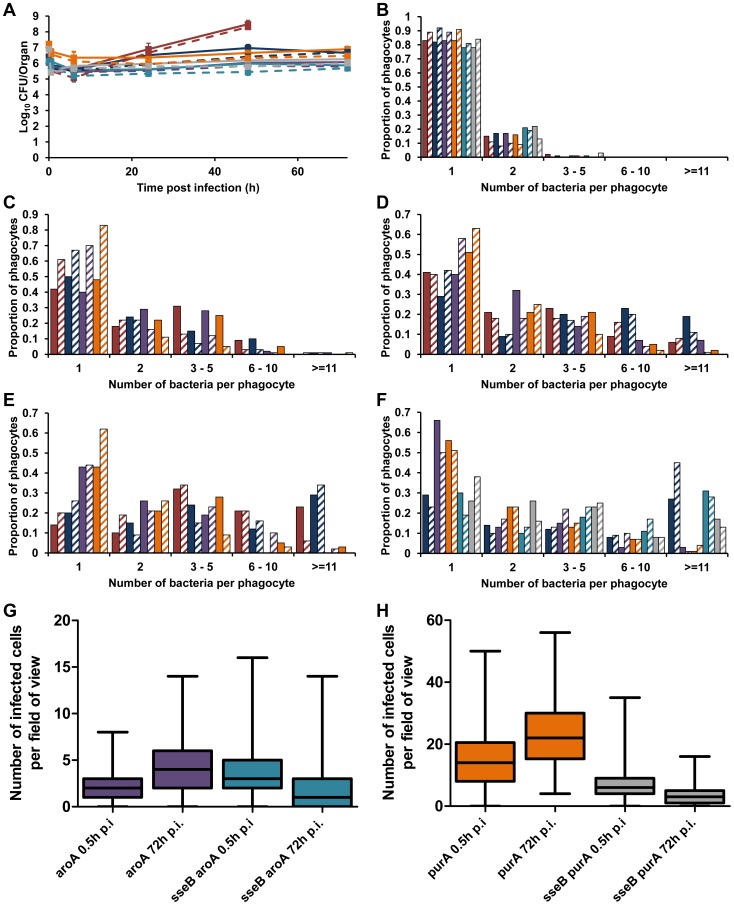
Similar net growth kinetics can be the result of very different intracellular infection dynamics. C57BL/6 mice were infected i.v. with ∼Log_10_ 6.2 CFU ( = 1.56×10^6^ CFU) of S12023, ∼Log_10_ 6.4 CFU ( = 2.64×10^6^ CFU) of S12023 *sseB*, ∼Log_10_ 6 CFU ( = 1.10×10^6^ CFU) of S12023 *aroA*, ∼Log_10_ 7.2 CFU ( = 1.69×10^7^ CFU) of S12023 *purA*, ∼Log_10_ 6.1 CFU ( = 1.37×10^6^ CFU) of S12023 *sseB aroA*, or ∼Log_10_ 6.9 CFU ( = 7.93×10^6^ CFU) of S12023 *sseB purA*. (A) Net bacterial numbers in livers (unbroken lines) and spleens (dotted lines) were determined between 0.5 and 72 h p.i. inclusive. The net bacterial growth of S12023 *sseB* was obtained from 4 mice per time point, S12023 (high input dose) was obtained from 3 mice per time point at 0.5, 6 and 24 h p.i. and 6 mice at the 48 h p.i. time point, S12023 *aroA*, S12023 *purA*, S12023 *sseB aroA* and S12023 *sseB purA* was obtained from 3 mice per time point (results are expressed as mean Log_10_ viable count ± standard deviation) [S12023 - red; S12023 *sseB* - blue; S12023 *aroA* - purple; S12023 *purA* – orange; S12023 *sseB aroA* – light blue; S12023 *sseB purA* - grey]. (B–F) The proportion of infected phagocytes relative to the number of bacteria contained within each phagocyte is shown for livers (solid bars) and spleens (diagonal shading) at (B) 0.5 h p.i.; (C) 6 h p.i.; (D) 24 h p.i.; (E) 48 h p.i. and (F) 72 h p.i.. The intracellular bacterial distributions of each strain were based on the counts obtained from 100 infected phagocytes per organ, per time point, from tissue obtained from 3 mice per time point. (G and H) Box and whisker plots showing the median, interquartile range and maximum and minimum number of infected cells per field-of-view in spleens at 0.5 h p.i. and 72 h p.i. for (G) S12023 *aroA* and S12023 *sseB aroA*, and (H) S12023 *purA* and S12023 *sseB purA*. The number of infected cells per field of view was obtained from 100 random fields from 3 mice for each strain at each time point.

To see whether these surprising intracellular bacterial growth dynamics underpin all attenuation of salmonellae in mice, we analyzed two other well-known and extensively described attenuated mutants. S12023 *aroA* (blocked in aromatic compound biosynthesis) and S12023 *purA* (deficient in purine metabolism) have very similar net growth characteristics to S12023 *sseB* (Figure 4A). Wild-type S12023 delivered at a similar dose to that used for the mutants exhibited low numbers of intracellular bacteria in livers and spleens throughout the infection (Figure 4B–D), despite exhibiting rapid net growth per organ. At 48 h p.i. these mice were very close to death, with large numbers of extracellular bacteria and necrotic organs; however, those cells that remained infected still had an intracellular density lower than that observed in heavily infected cells from mice infected with the S12023 *sseB* mutant at the same time point (Figure 4E). At each time point during the infection, the intracellular bacterial loads of the S12023 *aroA* and S12023 *purA* bacteria were heavily skewed towards low intracellular densities (Figure 4B–F, Figure S5 and Tables S1). Thus, similar net growth rates of salmonellae in organs can be derived from very different underlying intracellular growth dynamics (*e.g. aroA* mutant and *purA* mutant *vs sseB* mutant). We also generated double mutants, namely S12023 *sseB aroA* and S12023 *sseB purA*. These exhibited an increasing number of cells containing large numbers of intracellular bacteria during the infection (Figure 4B–F, Figure S5 and Table S1). Thus, the intracellular bacterial loads of the double mutants followed the *sseB* mutant pattern.

We then examined the distribution of infected cells in the tissues for the *aroA* and *purA* mutants and observed a small increase in the number of infected cells per field-of-view between 0.5 to 72 h p.i, for both S12023 *aroA* and S12023 *purA*, whereas for S12023 *sseB aroA* and S12023 *sseB purA* there was a small decrease in the number of infected cells per field-of-view over the same period of infection (Figures 4G and 4H, Figure S6 and Table S2). In other words the dispersion of the double mutants followed the *sseB* mutant pattern.

Finally, we co-infected mice with an *sseB* mutant and an *aroA* mutant simultaneously and at the same dose (Figure S7A). The intracellular bacterial loads of the different bacteria were the same as those seen when the two strains are injected individually, with very low intracellular densities observed for the *aroA* mutant and the characteristically higher densities for the *sseB* mutant (Figure S7B).

Overall the data show that very similar net growth kinetics can be the result of very different intracellular infection dynamics.

### ‘Clusters’ of SPI-2 mutant bacteria are not due to loss of SPI-2 T3SS-dependent cytotoxcity

Salmonellae induce host cell death during infection of cell cultures by several different mechanisms [29]. The exact contribution of each of these mechanisms to *in vivo* infection is far from clear. *In vitro* studies have demonstrated the involvement of SPI-2 T3SS effectors mediating cytotoxicity, namely SseL [30] and SpvB [31], [32]. Recently a model has been proposed, which suggests that *sseL* and *spv* genes promote host cell apoptosis, enabling the bacteria to be taken up by other cells resulting in further intracellular replication [29], [30].

We considered whether the reduced spread and increased number of SPI-2 mutant bacteria inside host cells could be due to the loss of SPI-2 T3SS-dependent cytotoxicity. We generated single mutants in *sseL*, *spvB* and an *sseL spvB* double mutant, and looked at intracellular bacterial numbers in the livers and spleens of C57BL/6 mice infected with these strains (data not shown). With none of these mutants did we observe the heavily infected cells characteristic of the SPI-2 T3SS mutants, suggesting that the loss of secretion of SseL and/or SpvB in the SPI-2 mutant is not responsible for the increased number of SPI-2 mutant bacteria inside host cells.

### Phagocyte NADPH oxidase inhibits *Salmonella* spread in the tissues in the absence of SPI-2 T3SS

In mammalian species, reactive oxygen radicals produced by the NADPH oxidase (Phox) are a major innate host defense mechanism against engulfed pathogens, including *Salmonella* [33], [34]. The dynamic consequences of the interplay between *Salmonella* SPI-2 and the NADPH oxidase at the single cell level *in vivo* are uncertain. gp91*phox* ^−/−^ mice lacking oxidase activity are fully susceptible to infection with SPI-2 mutants [33] and, in macrophages, a role has been ascribed to SPI-2 T3SS in the inhibition of the recruitment of the NADPH oxidase to the phagosome [35]–[37]. Recent studies have instead suggested a model in which *Salmonella* resistance relies on a range of detoxifying enzymes to cope with Phox-mediated oxidative stress [38].

**Figure 5:**
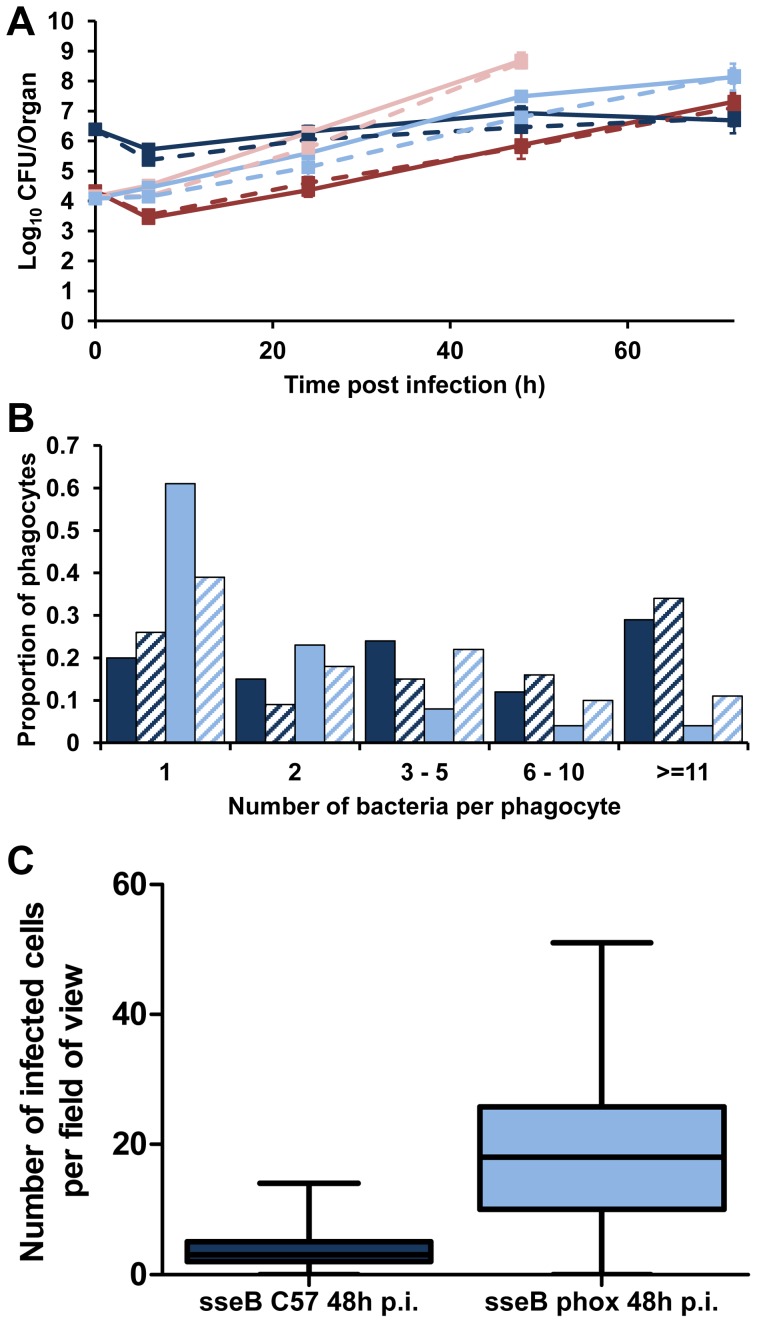
Phagocyte NADPH oxidase inhibits *Salmonella* spread in the tissues in the absence of SPI-2 T3SS. gp91*phox* ^−/−^ mice were infected i.v. with ∼Log_10_ 4.2 CFU ( = 1.45×10^4^ CFU) of S12023 or ∼Log_10_ 4.1 CFU ( = 1.21×10^4^ CFU) of S12023 *sseB*, C57BL/6 mice were infected i.v. with ∼Log_10_ 4.3 CFU ( = 2.15×10^4^ CFU) of S12023 or ∼Log_10_ 6.4 CFU ( = 2.45×10^6^ CFU) of S12023 *sseB*. (A) Net bacterial numbers in livers (unbroken lines) and spleens (dotted lines) were determined between 6 and 72 h p.i. inclusive (results are expressed as mean Log_10_ viable count ± standard deviation), the net bacterial growth of S12023 and S12023 *sseB* in gp91*phox* ^−/−^ mice was obtained from 3 mice per time point (the data for S12023 and S12023 *sseB* in C57BL/6 mice is reproduced from Figure 1F. S12023 in C57BL/6 mice – red; S12023 in gp91*phox* ^−/−^ mice – pink; S12023 *sseB* in C57BL/6 mice – dark blue; S12023 *sseB* in gp91*phox* ^−/−^ mice – light blue). (B) The proportion of infected phagocytes relative to the numbers of bacteria contained within each phagocyte for S12023 *sseB* in gp91*phox* ^−/−^ mice and S12023 *sseB* in C57BL/6 mice at 48 h p.i., based on the counts obtained from 100 infected phagocytes per organ, from tissue obtained from 3 mice per time point. (Livers – fully shaded; Spleens – diagonal shading). (C) Box and whisker plot showing the median, interquartile range and maximum and minimum number of infected cells per field-of-view for S12023 *sseB* in C57BL/6 mice and S12023 *sseB* in gp91*phox* ^−/−^ mice at 48 h p.i., from 100 random fields from 3 mice for each strain.

To explore the cellular dynamics that underpin the interplay between SPI-2 and Phox in *Salmonella*, we infected C57BL/6 and gp91*phox* ^−/−^ mice with S12023 wild-type and its *sseB* mutant (Figure 5A). As expected, the net growth rates of both the wild-type and the mutant were greater in the gp91*phox* ^−/−^ mice than in wild-type C57BL/6 mice confirming previous observations that SPI-2 mutants can grow rapidly in the tissues in the absence of a functional NADPH oxidase [33].

Next we observed the intracellular bacterial densities of S12023 *sseB* in gp91*phox* ^−/−^ mice at the 48 h p.i. time point, and compared them to our earlier data using the same bacterial mutant in C57BL/6 mice. Unexpectedly, we found that the *sseB* mutant in the gp91*phox* ^−/−^ mice was present at low numbers of bacteria per cell (Figure 5B, Figure S8 and Table S1), characteristic of wild-type *Salmonella* in a C57BL/6 mouse. Thus, in the absence of a functional NADPH oxidase the *sseB* mutant grew faster in the tissues, but did not accumulate within phagocytes.

Then we quantified the number of infected cells per field-of-view for the *sseB* mutant in C57BL/6 and gp91*phox* ^−/−^ mice throughout spleen tissues at 48 h p.i. (Figure 5C, Figure S9 and Table S2). The *sseB* mutant in the gp91*phox* ^−/−^ mice had formed many more infection foci than the *sseB* mutant in C57BL/6 mice despite there being similar total bacterial numbers in the tissues at this time (Figure 5A).

Taken together, these results suggest that in the absence of an active NADPH oxidase, SPI-2 T3SS becomes dispensable for the spread of *Salmonella* in the tissues as shown by increased numbers of infection foci and low intracellular densities of an *sseB* mutant in the gp91*phox* ^−/−^ mice. Conversely, when an active NADPH oxidase is present a SPI-2 T3SS mutant grows inside cells to high intracellular densities but appears to be impaired in tissue spread and formation of new infection foci. The fact that an *sseB* mutant can escape from cells in the gp91*phox* ^−/−^ mice also indicates that SPI-2 independent mechanisms can mediate escape from cells and that these as yet unidentified mechanisms/effectors are normally under the inhibitory effects of the NADPH oxidase in the absence of SPI-2. These results suggest a new interplay between SPI-2 T3SS and innate immunity in the dynamics of within-host bacterial growth and spread. Where NADPH-mediated mechanisms prevent bacterial escape from cells and at the same time exert bactericidal/bacteriostatic activity on the intracellular bacteria. However, we cannot rule out the possibility that the dynamics in the gp91*phox* ^−/−^ mice are an artifact of infection of the mutant mice with the mutant bacteria and may be proceeding by mechanisms different to natural murine infection.

## Discussion

We have shown that intracellular *Salmonella* infection dynamics *in vivo* are dramatically altered by deletion of the SPI-2 T3SS in an unexpected way. Wild-type bacteria are present in low numbers per cell on average, and the rapid net growth of these bacteria in tissues coincides with escape from the intracellular environment and dispersal to new cells where new infection foci are established. On the other hand, mutants lacking SPI-2 T3SS seem unable to escape from the infected cell and by inference are therefore unable to disperse through the tissues. What is more, the SPI-2 T3SS mutants are able to grow to high numbers within the intracellular environment, which is not what has been reported as happening in many studies of infected macrophages *in vitro*. The observation that two variants of the *Salmonella sseB* mutant, which can be labelled either red or green, segregate to different infected cells upon simultaneous infection into the same animal provides strong evidence that in systemic *Salmonella* infections each infected cell and each multicellular infection focus is the product of the clonal expansion of a single founder bacterium. This also excludes the possibility that the mutant bacteria have accumulated intracellularly by phagocytosis. Therefore, even if a proportion of the bacteria were dead or dormant at the time of observation the high intracellular bacterial numbers observed in animals infected with the *sseB* mutants show that these mutants can and do grow to high numbers per cell as a result of intracellular division. Our results call into question the usefulness of *in vitro* systems for studying the natural history and dynamics of *Salmonella* infection of cells when these methods are used in isolation and not validated against what actually happens in the infected animal.

Another important observation from our study is that very similar net growth rates in organs can be derived from very different underlying intracellular growth dynamics. For example, slow growth of salmonellae per organ, typical of bacteria that are currently being trialed in humans as attenuated vaccine strains, can be generated by highly dispersed infections with low numbers of bacteria per cell, as is the case for *aroA* mutant bacteria, or by relatively non-dispersed infections with high numbers of bacteria per cell, as we have shown here is the case for SPI-2 mutants.

That the phagocyte oxidase in involved in the restraint of spread of the salmonellae is a new concept, and suggests that this important innate immunity mechanism hampers bacterial escape from cells and at the same time (our previous work [33]–[35]) exerts antimicrobial functions on the intracellular bacteria.

Very high quality science has been published, concerning the dynamics of infectious disease spread through communities of people or animals, but much less work has been done to understand infectious disease dynamics within the host. Many conclusions about how infectious agents work are based on experiments in isolated monocultures of cells or in somewhat crude experiments in whole animals, where gross read-outs are used to try to capture what is a complex underlying process. Understanding this complex process at a more detailed level in whole animals is the next major challenge for infectious disease biologists, and is required if intervention strategies to prevent and cure infectious diseases are to be improved and targeted effectively.

## Materials and Methods

### Ethics statement

All animals were handled in strict accordance with good animal practice as defined by the relevant international (Directive of the European Parliament and of the Council on the protection of animals used for scientific purposes, Brussels 543/5) and local (Department of Veterinary Medicine, University of Cambridge) animal welfare guidelines. All animal work was approved by the ethical review committee of the University of Cambridge and was licensed by the UK Government Home Office under the Animals (Scientific Procedures) Act 1986.

### Bacterial strains and growth conditions

We used *Salmonella enterica* serovar Typhimurium strain NCTC S12023 (wild-type) (identical to ATCC 14028s) and mutant strains S12023 *ssaV* [39], S12023 *sseB* [5], S12023 *sseB*(psseB) [5], S12023 *sseL* [30], S12023 *aroA* (this study), S12023 *purA* (this study), S12023 *sseB aroA* (this study), S12023 *sseB purA* (this study), S12023 *spiC* (this study), S12023 *ssaM* (this study), S12023 *spvB* (this study) and S12023 *sseL spvB* (this study). We used *S*. Typhimurium C5, a highly virulent strain with an intravenous (i.v.) LD_50_ of <10 CFU in innately susceptible mice [40] and mutant strain C5 *ssaV* (this study). We used *S*. Typhimurium SL5559 and SL5560 which are sister transductants of *S.* Typhimurium C5 that differ only in O antigen type allowing their differential identification after immunostaining [41], and mutant strains SL5559 *aroA* (this study), SL5559 *sseB* (this study) and SL5560 *sseB* (this study). We used *S.* Typhimurium SL1344, a virulent wild-type strain which has an LD_50_ by the i.v. route of <20 CFU for innately susceptible mice [42], and mutant strain SL1344 *ssaV* (this study). Preparation of electrocompetent *Escherichia coli* and *S. enterica* cells and transformations were performed as previously described [43]. Bacteria were grown on Luria-Bertani (LB) medium. Media were supplemented with the appropriate antibiotic for selection (ampicillin 100 µg/ml, kanamycin 50 µg/ml or chloramphenicol 10 µg/ml). *In vitro* growth rates of *Salmonella* strains in LB broth were determined by both optical density and viable count.

### Recombinant DNA techniques

Standard methods were used for molecular cloning [44]. Chromosomal and plasmid DNA purifications, and routine DNA modifications including restriction endonuclease digestion of DNA, modifications of DNA and ligations were carried out using commercial kits and supplies according to the manufacturers' instructions (QIAGEN, Crawley, UK; Promega, Southampton, UK; Invitrogen, Paisley, UK; Roche, Lewes, UK; New England Biolabs, Hitchin, UK). DNA concentration and purity were measured using a Nanodrop ND-1000 spectrophotometer. PCR primers were designed using Primer3 (http://frodo.wi.mit.edu/) and purchased from Sigma (Sigma-Genosys, UK). PCRs were performed in 25 µl reaction volumes in 0.2 ml Eppendorf tubes in a Perkin Elmer Gene Amp 2400 thermal cycler. Reactions contained 200 µM dNTPs, 2 mM Mg^2+^, 0.01 volumes of Proof Start DNA polymerase (QIAgen; 2.5 U µl^−1^), 0.1 volumes polymerase buffer (10×), 1 µM forward and reverse primers and template DNA (∼50 ng plasmid DNA or ∼100 ng chromosomal DNA). Thermal cycler conditions were 94°C for 10 min, then 35 cycles of 94°C for 1 min, 55°C for 1 min and 72°C for 1 min, followed by a final extension at 72°C for 10 min.

### Generation of *S. enterica* mutants

Mutants were generated using a modification of the ET-cloning procedure [45], [46] as previously described [47]. PCR was used to amplify the chloramphenicol resistance cassette from pACYC184 [48] or the kanamycin resistance cassette from pACYC177 [48] with 5′ and 3′ 60 bp homology arms complementary to the flanking regions of the gene to be deleted. Approx 1 µg of linear PCR product was used for integration onto the chromosome using a modification of the Lambda Red method [49], as previously detailed [14]. Transformants were selected by plating onto media containing chloramphenicol or kanamycin. Screening for loss of the pBADλred helper plasmid was essentially as previously described [50], using MAST ID Intralactam circles (MAST Diagnostics, Bootle Merseyside, UK) to screen for the absence of beta-lactamase in bacterial colonies. (Further details of mutant constructions are provided in Supporting Information – Protocol S1).

### Mouse infections

Sex- and aged-matched 9–12 week old C57BL/6 mice (Harlan Olac Ltd) and gp91*phox* ^−/−^ mice (bred at the Wellcome Trust Sanger Institute, Hinxton, Cambridge, United Kingdom) were infected by intravenous (i.v.) injection of bacterial suspensions in a volume of 0.2 ml. Bacterial cultures were grown from single colonies in 10 ml LB broth incubated overnight without shaking at 37°C, then diluted in phosphate buffered saline (PBS) to the appropriate concentration for inoculation. Inocula were enumerated by plating dilutions onto LB agar plates. Mice were killed by cervical dislocation and the livers and spleens were aseptically removed and homogenized in sterile water using a Colworth Stomacher 80. The resulting homogenate was diluted in a 10-fold series in PBS and LB agar pour plates were used to enumerate viable bacteria.

### Immunostaining for microscopy

Half of each organ was fixed overnight in 4% paraformaldehyde diluted in PBS, washed for 90 min in three changes of PBS and then immersed in 20% sucrose in PBS for 16 h at 4°C before being embedded in Optimal Cutting Temperature (OCT) (Raymond A Lamb Ltd, Eastbourne, U.K.) in cryomoulds (Park Scientific, Northampton, U.K.). Samples were frozen and stored at −80°C. 30 µm sections were cut, blocked and permeabilized for 10 min in a solution containing 10% normal goat serum and 0.02% Saponin in PBS (Sigma, Poole, UK). Subsequently sections were incubated with primary antibodies in permeabilizing solution, washed in PBS then incubated with secondary antibodies and observed using a fluorescence microscope (Leica DM6000B), or a confocal laser scanning microscope (Leica TCS SP5). Primary antibodies used in this study were: a 1∶1000 dilution (Figures 1B, 1C, 1D, 1E, 2A, 2B, 2C, 3A, 3B, 4B, 4C, 4D, 4E, 4F, 4G, 4H, 5A, 5B, 5C, S3B and S3C), a 1∶100 dilution (Figures 3B and S7B) of rat anti-mouse CD18^+^ monoclonal antibody (clone M18/2, BD Pharmingen); a 1∶500 dilution (Figures 1B, 1C, 1D, 1E, 1G, 2A, 2B, 2C, 3A, 3B, 4B, 4C, 4D, 4E, 4F, 4G, 4H, 5B, 5C, S3B and S3C) of rabbit anti-LPS O4 agglutinating antiserum (Remel Europe Ltd); a 1∶500 dilution (Figures 3C and S7B) of rabbit anti-LPS O9 agglutinating serum (Remel Europe Ltd). Secondary antibodies used in this study were: a 1∶100 dilution of Alexa Fluor 568-conjugated goat anti-rat antibody (Invitrogen-Molecular Probes, U.K.) and a 1∶1000 dilution of Alexa Fluor 488-conjugated goat anti-rabbit antibody (Invitrogen-Molecular Probes, U.K.) (Figures 1B, 1C, 1D, 1E, 1G, 2A, 2B, 2C, 3A, 3B, 4B, 4C, 4D, 4E, 4F, 4G, 4H, 5B, 5C, S3B and S3C). All sections were mounted onto Vectabond-treated glass slides (Vector Laboratories Ltd.) using Vectashield containing DAPI (Vector Laboratories Ltd.) for fluorescence microscopy and Fluoromount-G (SouthernBiotech) for confocal microscopy. Intracellular bacterial distributions were counted by eye, from tissue obtained from multiple mice per group, as indicated in each Figure legend.

### Statistical analysis

All data analysis was produced using the open-source R statistical language [51]. The MCMC routines were written in C and C++ utilizing the GNU GSL library [52]. The R package “coda” [53], was used to read in and summarize the output from the MCMC runs. Color palettes in the plots were obtained from the “RColorBrewer” package [54]. Data in the tables are given to 2 significant figures. (Further modeling detail is provided in Supporting Information – Protocol S1).

## Supporting Information

Figure S1 **Posterior means and 95% credible intervals of intracellular bacterial distributions (0.5 h p.i and 72 h p.i.).** (A to F) Barplots showing the proportions of infected cells in each bacterial load category (1, 2, 3–5, 6–10 and ≥11) aggregated across mice but stratified by bacterial strain [A and B, S12023 wild-type; C and D, S12023 *sseB*; and E and F, S12023 *sseB*(psseB)], organ (liver and spleen) and time post infection (0.5 h p.i. and 72 h p.i.). The red bars correspond to the S12023 infections for (A) livers and (B) spleens, the blue bars to S12023 *sseB* infections for (C) livers and (D) spleens, and the green bars to S12023 *sseB*(psseB) infections for (E) livers and (F) spleens. The darker shades correspond to the 0.5 h p.i. time point and the lighter shades to the 72 h p.i. time point. The marginal distributions for the probability of belonging to each group obtained from a hierarchical Bayesian ordinal regression model are represented by the posterior means and 95% credible intervals (shown by the points and error lines).
(TIF)

Figure S2 **Fitted negative binomial distributions against observed number of infected cells per field-of-view [wild-type,** ***sseB*** **,** ***sseB*** **(psseB)].** (A) S12023 at 72 h p.i., (B) S12023 *sseB* at 0.5 h p.i., (C) S12023 *sseB* at 72 h p.i., (D) S12023 *sseB*(psseB) at 72 h p.i.
(TIF)

Figure S3 **SpiC is required for** ***S*** **. Typhimurium to disperse in the tissues.** C57BL/6 mice were infected i.v. with ∼Log_10_ 6.3 CFU ( = 1.89×10^6^ CFU) of S12023 *spiC*. (A) Net bacterial numbers in livers (unbroken line) and spleens (dotted line) were determined at time points between 0.5 and 72 h p.i. inclusive, from 4 mice per time point (results are expressed as mean Log_10_ viable count ± standard deviation). (B) The proportion of infected phagocytes relative to the numbers of bacteria contained within each phagocyte at 0.5 and 72 h p.i., based on the counts obtained from 100 infected phagocytes per organ, per time point, from tissue obtained from 4 mice per time point (Livers – fully shaded; Spleens – diagonal shading). (C) Box and whisker plot showing the median, interquartile range and maximum and minimum number of infected cells per field-of-view for S12023 *spiC* in spleens at 0.5 and 72 h p.i. obtained from 100 random fields from 4 mice at each time point. (D and E) Fitted negative binomial distributions against the observed data for the number of S12023 *spiC* infected cells per field of view, stratified by time (D) 0.5 h p.i. and (E) 72 h p.i.
(TIF)

Figure S4 **Posterior means and 95% credible intervals of intracellular bacterial distributions of S12023** ***sseB*** **.** (A and B) Barplots showing the proportions of infected cells in each bacterial load category (1, 2, 3–5, 6–10 and ≥11) aggregated across all mice but stratified by organ (A, liver and B, Spleen) and time (0.5, 6, 24, 48 and 72 h p.i.). The shading gets lighter as time progresses. The marginal distributions for the probability of belonging to each group obtained from a hierarchical Bayesian ordinal regression model are represented by the posterior means and 95% credible intervals (shown by the points and error lines).
(TIF)

Figure S5 **Posterior means and 95% credible intervals of intracellular bacterial distributions of** ***aroA*** **and** ***purA*** **mutants.** (A to D) Barplots showing the proportions of infected cells in each bacterial load category (1, 2, 3–5, 6–10 and ≥11) aggregated across all mice but stratified by strain (S12023 *sseB*, S12023 *aroA*, S12023 *purA*, S12023 *sseB aroA* and S12023 *sseB purA*), organ (A and C, livers and B and D, spleens) and time (A and B, 0.5 h p.i. and C and D, 72 h p.i.). The blue bars correspond to S12023 *sseB*, the purple bars to S12023 *aroA*, the orange bars to S12023 *purA*, the light blue bars to S12023 *sseB aroA* and the grey bars to S12023 *sseB purA*. The marginal distributions for the probability of belonging to each group obtained from a hierarchical Bayesian ordinal regression model are represented by the posterior means and 95% credible intervals (shown by the points and error lines).
(TIF)

Figure S6 **Fitted negative binomial distributions against observed number of infected cells per field-of-view (** ***aroA*** **and** ***purA*** **).** (A) S12023 *aroA* at 0.5 h p.i., (B) S12023 *aroA* at 72 h p.i., (C) S12023 *purA* at 0.5 h p.i., (D) S12023 *purA* at 72 h p.i., (E) S12023 *sseB aroA* at 0.5 h p.i., (F) S12023 *sseB aroA* at 72 h p.i., (G) S12023 *sseB purA* at 0.5 h p.i., (H) S12023 *sseB purA* at 72 h p.i..
(TIF)

Figure S7 **Intracellular bacterial distributions of SL5559** ***aroA*** **and SL5560** ***sseB*** **in the organs of infected mice.** (A and B) C57BL/6 mice were infected i.v. with ∼Log_10_ 5.9 colony forming units (CFU) ( = 7.33×10^5^ CFU) of SL5559 *aroA* and ∼Log_10_ 6.0 CFU ( = 9.20×10^5^ CFU) of SL5560 *sseB* bacteria *via* a single injection. (A) Net bacterial numbers in livers (unbroken line) and spleens (dotted line) were determined between 0.5 to 72 h p.i. (results are expressed as mean Log_10_ viable count ± standard deviation, from 3 mice per group, SL5559 *aroA* - purple; SL5560 *sseB* - blue). (B) Representative fluorescence micrograph of *Salmonella* SL5559 *aroA* and SL5560 *sseB* mutants (inoculated into the same animal *via* a single injection) within phagocytes in an infected spleen of a C57BL/6 mouse at 72 h p.i.. SL5559 *aroA* (green), SL5560 *sseB* (red), nucleic acid is stained with DAPI (blue). Scale bar, 75 µm.
(TIF)

Figure S8 **Posterior means and 95% credible intervals of intracellular** ***sseB*** **distributions in wild-type and gp91** ***phox*** **^−/−^ mice.** (A to D) Barplots showing the proportions of infected cells in each bacterial load category (1, 2, 3–5, 6–10 and ≥11) aggregated across mice but stratified by bacterial strain [A and B, S12023 *sseB* in C57BL/6 mice; C and D, S12023 *sseB* in gp91*phox* ^−/−^ mice], organ (liver and spleen) (data in table S18). The red bars correspond to the S12023 *sseB* infections in C57BL/6 mice for (A) livers and (B) spleens, the blue bars to S12023 *sseB* infections in gp91*phox* ^−/−^ mice for (C) livers and (D) spleens. The marginal distributions for the probability of belonging to each group obtained from a hierarchical Bayesian ordinal regression model are represented by the posterior means and 95% credible intervals (shown by the points and error lines).
(TIF)

Figure S9 **Fitted negative binomial distributions against observed number of infected cells per field-of-view, by mouse genotype.** (A) S12023 *sseB* at 48 h p.i. in C57BL/6 mice, (B) S12023 *sseB* at 48 h p.i. in gp91*phox* ^−/−^ mice.
(TIF)

Protocol S1 **Details of** ***S. enterica*** **mutant generation, model constructions and statistical analyses.**
(DOCX)

Table S1 **Posterior means and 95% credible intervals for proportional odds ratios from hierarchical Bayesian ordinal regression.**
(DOCX)

Table S2 **Differences between mean number of infected cells per field-of-view, from hierarchical Bayesian negative binomial regression.**
(DOCX)

Table S3 **Primer sequences used in this study.**
(DOCX)
