# Supplementary figures and images for: Attenuated Salmonella Typhimurium Lacking the Pathogenicity Island-2 Type 3 Secretion System Grow to High Bacterial Numbers inside Phagocytes in Mice
Source: PLoS Pathog. 2012 Dec 6;8(12):e1003070. doi: 10.1371/journal.ppat.1003070 (PMC3516571; doi:10.1371/journal.ppat.1003070)

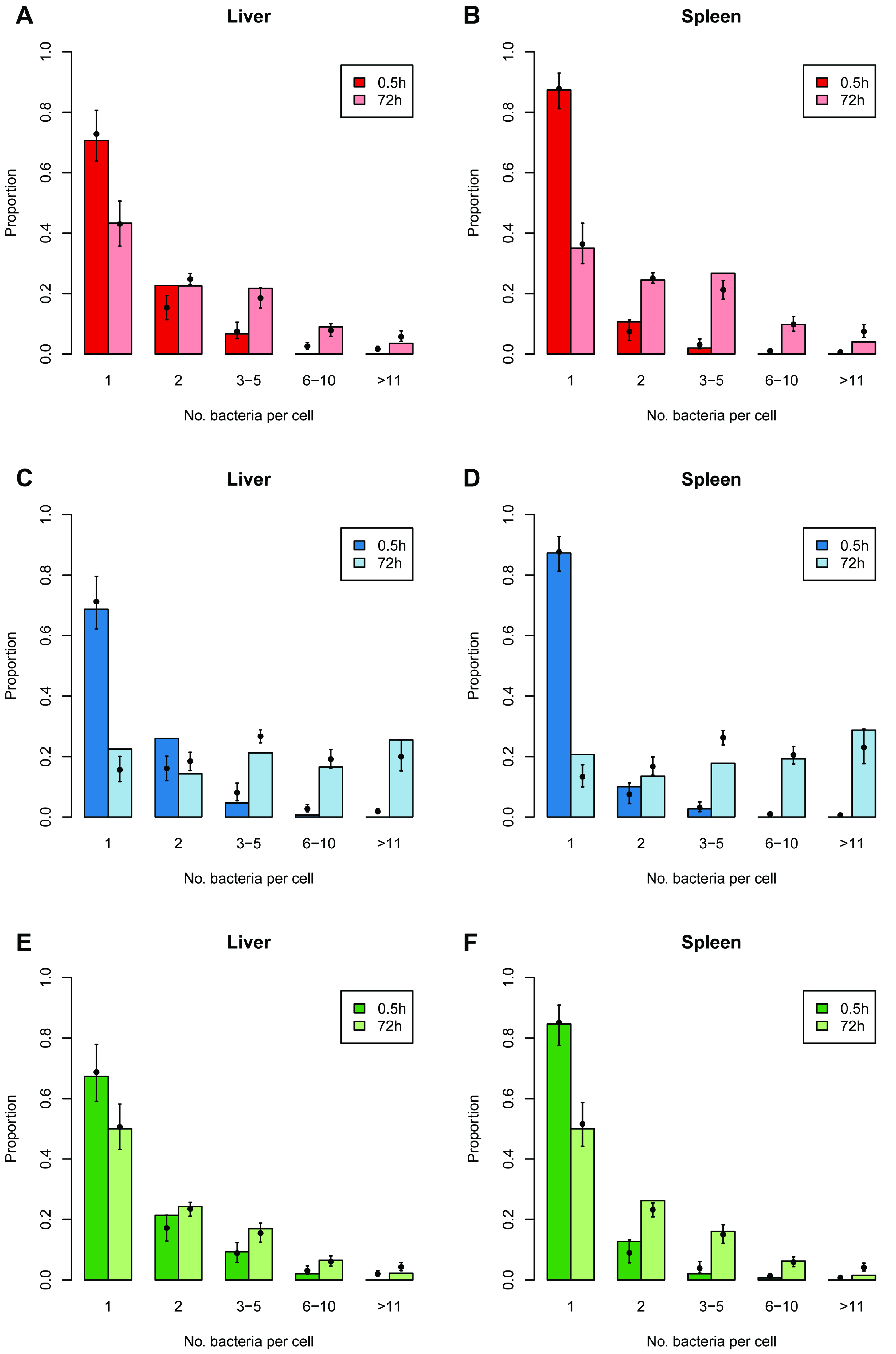

Supplement: Figure S1 — Posterior means and 95% credible intervals of intracellular bacterial distributions (0.5 h p.i and 72 h p.i.). (A to F) Barplots showing the proportions of infected cells in each bacterial load category (1, 2, 3–5, 6–10 and ≥11) aggregated across mice but stratified by bacterial strain [A and B, S12023 wild-type; C and D, S12023 sseB; and E and F, S12023 sseB(psseB)], organ (liver and spleen) and time post infection (0.5 h p.i. and 72 h p.i.). The red bars correspond to the S12023 infections for (A) livers and (B) spleens, the blue bars to S12023 sseB infections for (C) livers and (D) spleens, and the green bars to S12023 sseB(psseB) infections for (E) livers and (F) spleens. The darker shades correspond to the 0.5 h p.i. time point and the lighter shades to the 72 h p.i. time point. The marginal distributions for the probability of belonging to each group obtained from a hierarchical Bayesian ordinal regression model are represented by the posterior means and 95% credible intervals (shown by the points and error lines). (TIF) [file ppat.1003070.s001.tif]

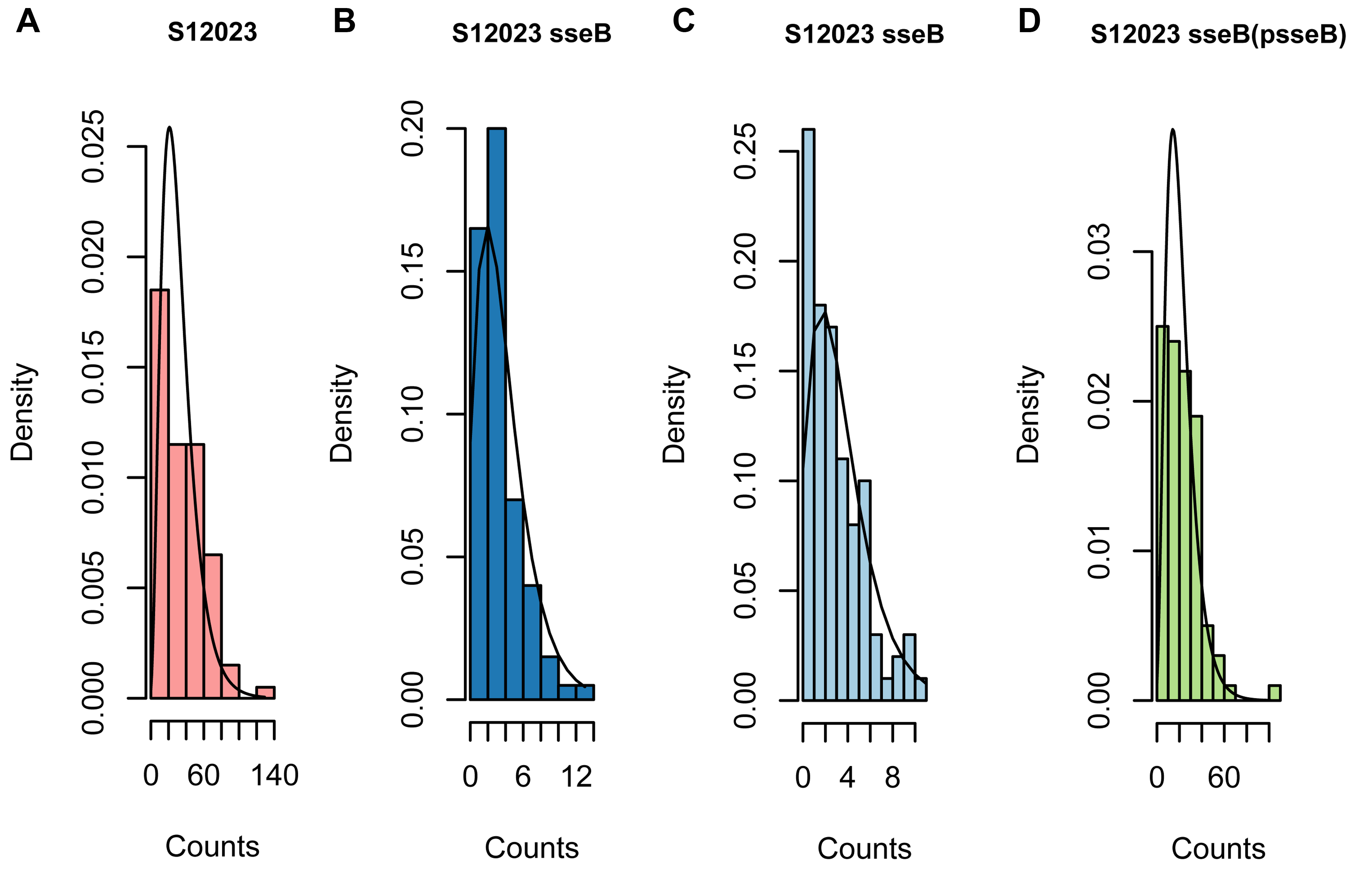

Supplement: Figure S2 — Fitted negative binomial distributions against observed number of infected cells per field-of-view [wild-type, sseB , sseB (psseB)]. (A) S12023 at 72 h p.i., (B) S12023 sseB at 0.5 h p.i., (C) S12023 sseB at 72 h p.i., (D) S12023 sseB(psseB) at 72 h p.i. (TIF) [file ppat.1003070.s002.tif]

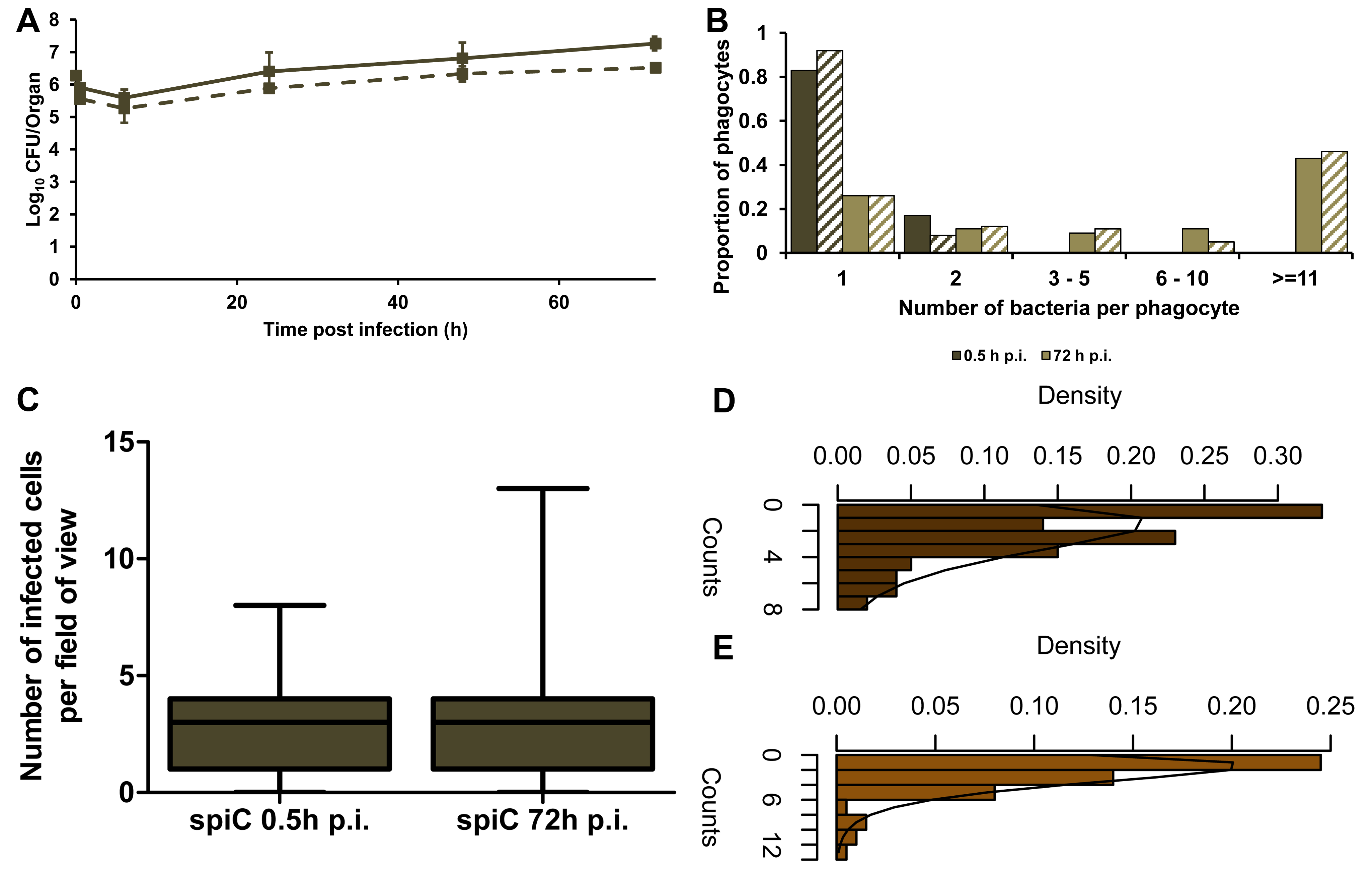

Supplement: Figure S3 — SpiC is required for S . Typhimurium to disperse in the tissues. C57BL/6 mice were infected i.v. with ∼Log10 6.3 CFU ( = 1.89×10^6^ CFU) of S12023 spiC. (A) Net bacterial numbers in livers (unbroken line) and spleens (dotted line) were determined at time points between 0.5 and 72 h p.i. inclusive, from 4 mice per time point (results are expressed as mean Log10 viable count ± standard deviation). (B) The proportion of infected phagocytes relative to the numbers of bacteria contained within each phagocyte at 0.5 and 72 h p.i., based on the counts obtained from 100 infected phagocytes per organ, per time point, from tissue obtained from 4 mice per time point (Livers – fully shaded; Spleens – diagonal shading). (C) Box and whisker plot showing the median, interquartile range and maximum and minimum number of infected cells per field-of-view for S12023 spiC in spleens at 0.5 and 72 h p.i. obtained from 100 random fields from 4 mice at each time point. (D and E) Fitted negative binomial distributions against the observed data for the number of S12023 spiC infected cells per field of view, stratified by time (D) 0.5 h p.i. and (E) 72 h p.i. (TIF) [file ppat.1003070.s003.tif]

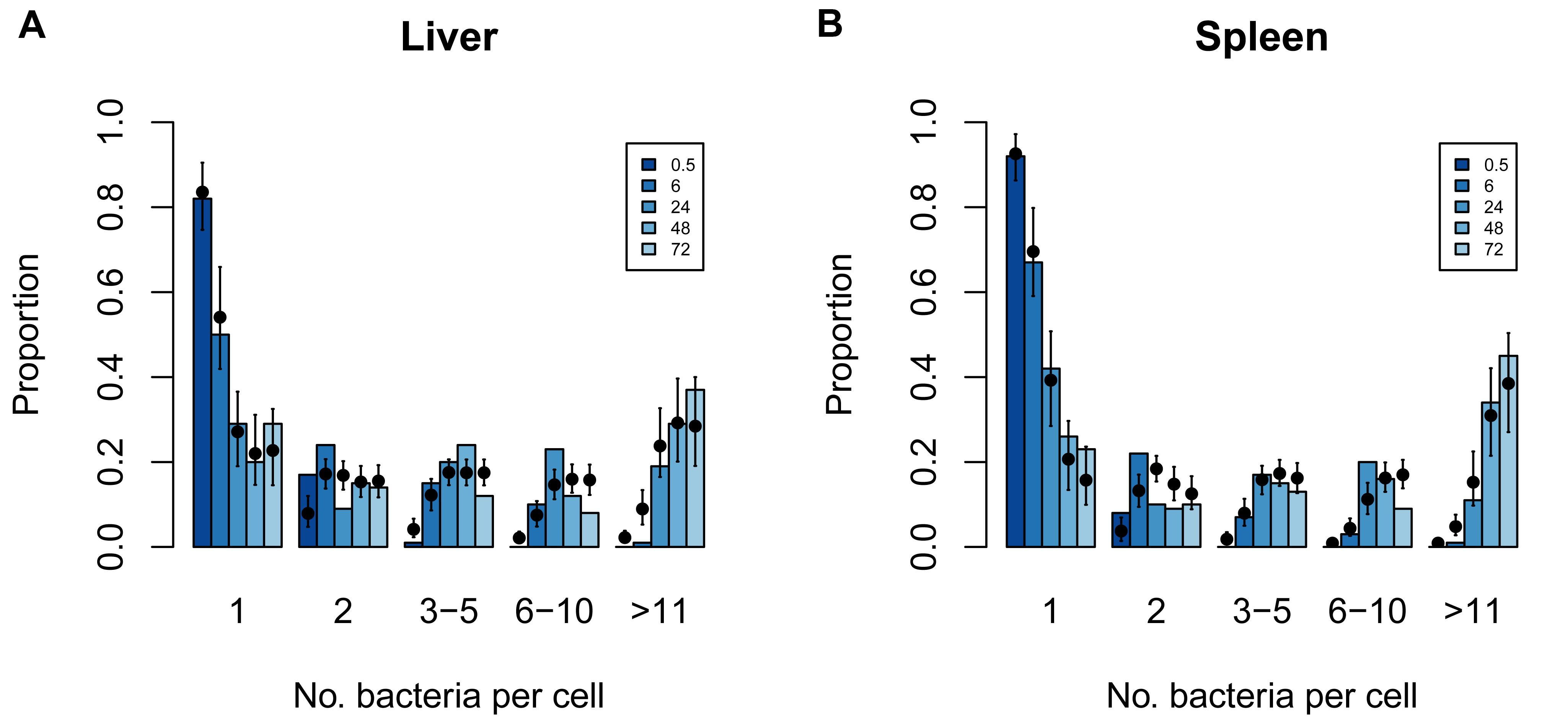

Supplement: Figure S4 — Posterior means and 95% credible intervals of intracellular bacterial distributions of S12023 sseB . (A and B) Barplots showing the proportions of infected cells in each bacterial load category (1, 2, 3–5, 6–10 and ≥11) aggregated across all mice but stratified by organ (A, liver and B, Spleen) and time (0.5, 6, 24, 48 and 72 h p.i.). The shading gets lighter as time progresses. The marginal distributions for the probability of belonging to each group obtained from a hierarchical Bayesian ordinal regression model are represented by the posterior means and 95% credible intervals (shown by the points and error lines). (TIF) [file ppat.1003070.s004.tif]

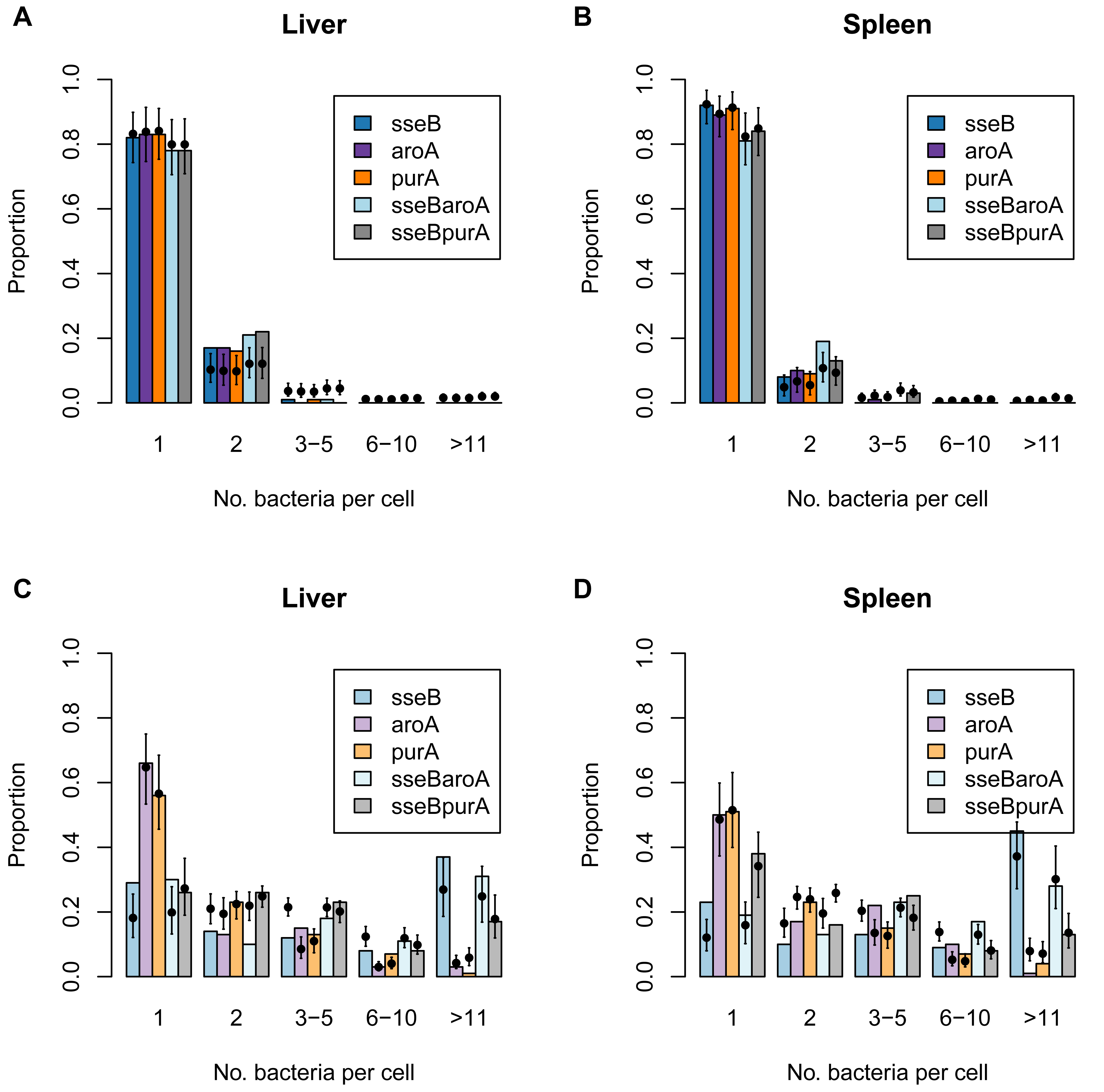

Supplement: Figure S5 — Posterior means and 95% credible intervals of intracellular bacterial distributions of aroA and purA mutants. (A to D) Barplots showing the proportions of infected cells in each bacterial load category (1, 2, 3–5, 6–10 and ≥11) aggregated across all mice but stratified by strain (S12023 sseB, S12023 aroA, S12023 purA, S12023 sseB aroA and S12023 sseB purA), organ (A and C, livers and B and D, spleens) and time (A and B, 0.5 h p.i. and C and D, 72 h p.i.). The blue bars correspond to S12023 sseB, the purple bars to S12023 aroA, the orange bars to S12023 purA, the light blue bars to S12023 sseB aroA and the grey bars to S12023 sseB purA. The marginal distributions for the probability of belonging to each group obtained from a hierarchical Bayesian ordinal regression model are represented by the posterior means and 95% credible intervals (shown by the points and error lines). (TIF) [file ppat.1003070.s005.tif]

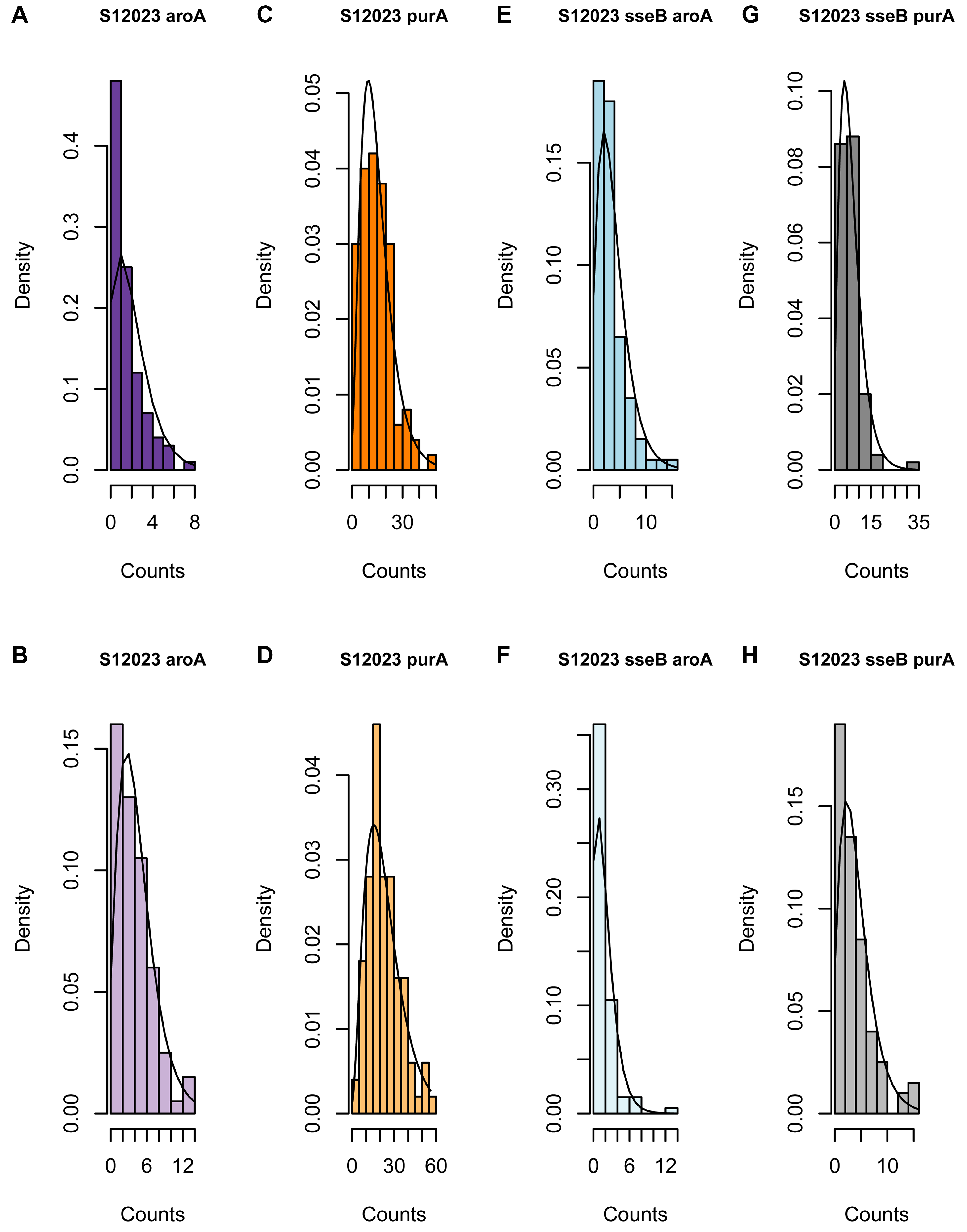

Supplement: Figure S6 — Fitted negative binomial distributions against observed number of infected cells per field-of-view ( aroA and purA ). (A) S12023 aroA at 0.5 h p.i., (B) S12023 aroA at 72 h p.i., (C) S12023 purA at 0.5 h p.i., (D) S12023 purA at 72 h p.i., (E) S12023 sseB aroA at 0.5 h p.i., (F) S12023 sseB aroA at 72 h p.i., (G) S12023 sseB purA at 0.5 h p.i., (H) S12023 sseB purA at 72 h p.i.. (TIF) [file ppat.1003070.s006.tif]

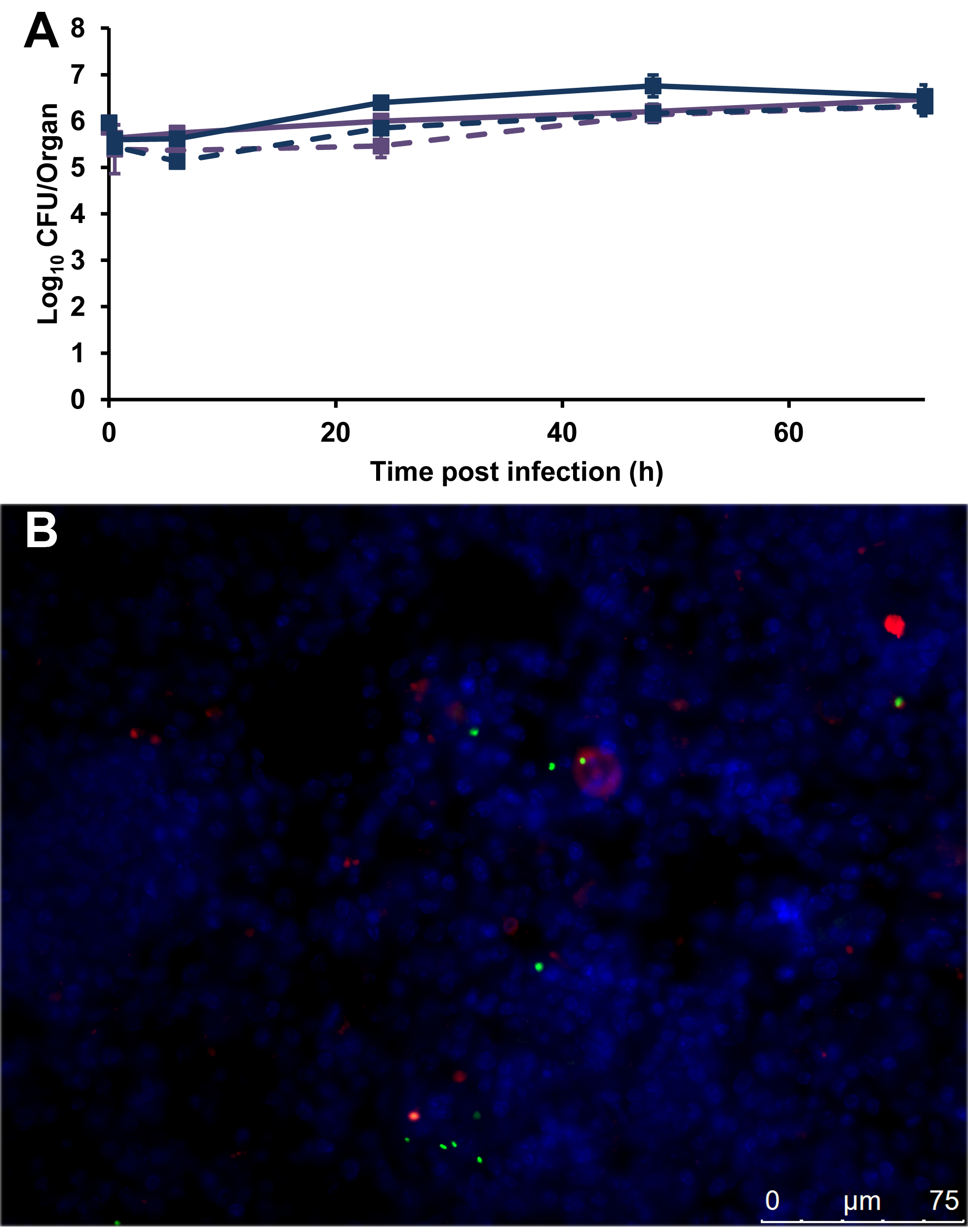

Supplement: Figure S7 — Intracellular bacterial distributions of SL5559 aroA and SL5560 sseB in the organs of infected mice. (A and B) C57BL/6 mice were infected i.v. with ∼Log10 5.9 colony forming units (CFU) ( = 7.33×10^5^ CFU) of SL5559 aroA and ∼Log10 6.0 CFU ( = 9.20×10^5^ CFU) of SL5560 sseB bacteria via a single injection. (A) Net bacterial numbers in livers (unbroken line) and spleens (dotted line) were determined between 0.5 to 72 h p.i. (results are expressed as mean Log10 viable count ± standard deviation, from 3 mice per group, SL5559 aroA - purple; SL5560 sseB - blue). (B) Representative fluorescence micrograph of Salmonella SL5559 aroA and SL5560 sseB mutants (inoculated into the same animal via a single injection) within phagocytes in an infected spleen of a C57BL/6 mouse at 72 h p.i.. SL5559 aroA (green), SL5560 sseB (red), nucleic acid is stained with DAPI (blue). Scale bar, 75 µm. (TIF) [file ppat.1003070.s007.tif]

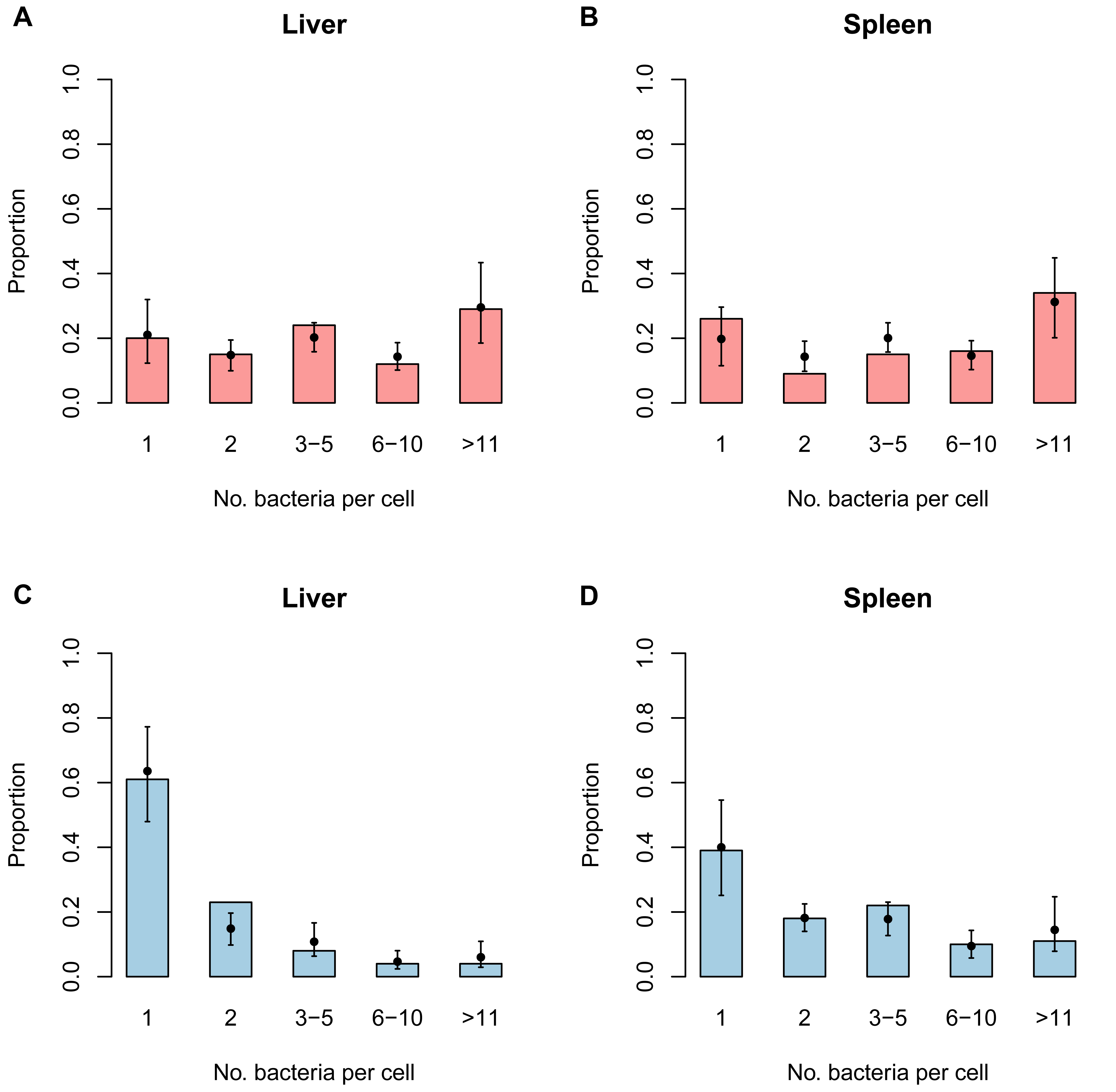

Supplement: Figure S8 — Posterior means and 95% credible intervals of intracellular sseB distributions in wild-type and gp91 phox −/− mice. (A to D) Barplots showing the proportions of infected cells in each bacterial load category (1, 2, 3–5, 6–10 and ≥11) aggregated across mice but stratified by bacterial strain [A and B, S12023 sseB in C57BL/6 mice; C and D, S12023 sseB in gp91phox −/− mice], organ (liver and spleen) (data in table S18). The red bars correspond to the S12023 sseB infections in C57BL/6 mice for (A) livers and (B) spleens, the blue bars to S12023 sseB infections in gp91phox −/− mice for (C) livers and (D) spleens. The marginal distributions for the probability of belonging to each group obtained from a hierarchical Bayesian ordinal regression model are represented by the posterior means and 95% credible intervals (shown by the points and error lines). (TIF) [file ppat.1003070.s008.tif]

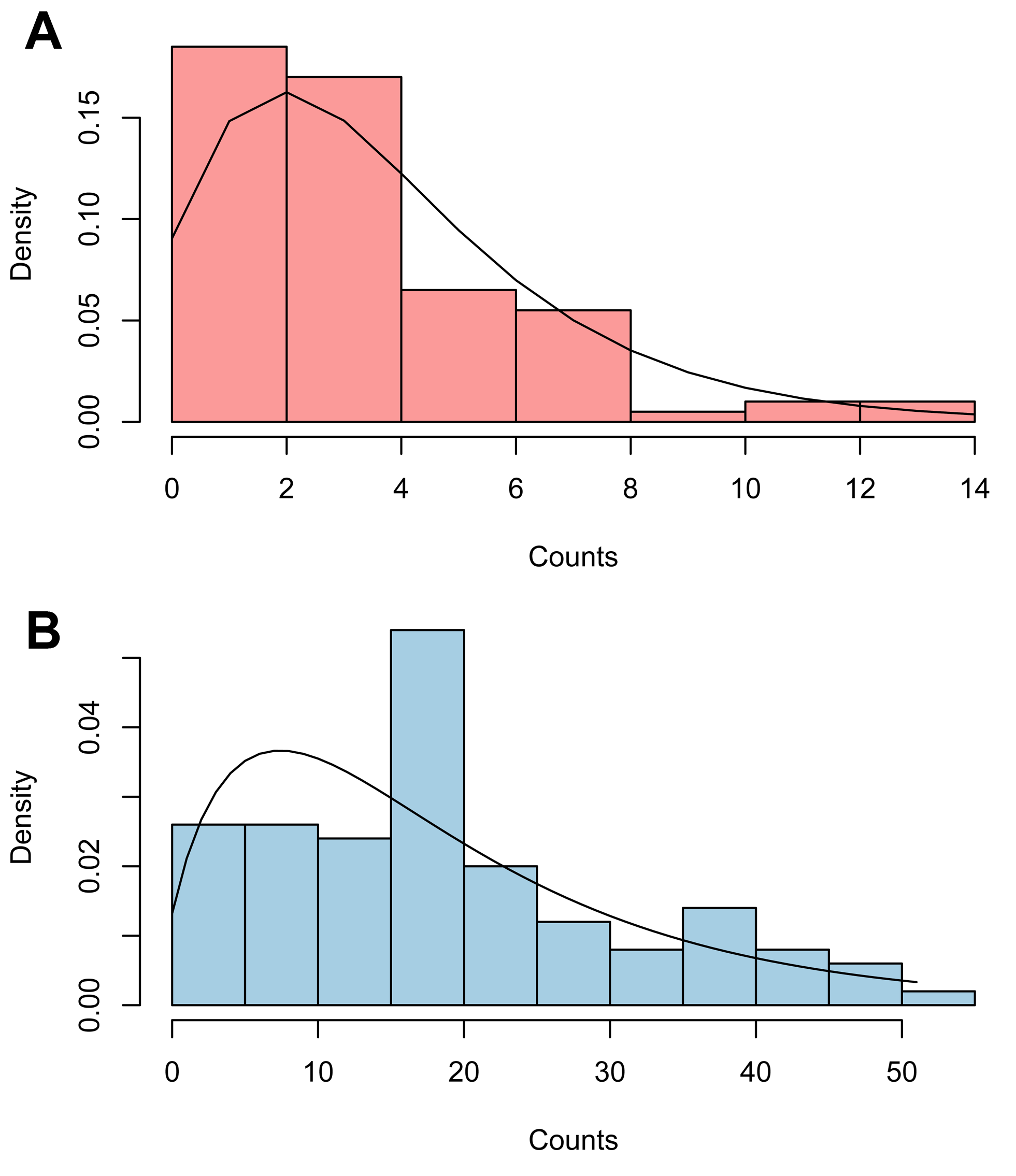

Supplement: Figure S9 — Fitted negative binomial distributions against observed number of infected cells per field-of-view, by mouse genotype. (A) S12023 sseB at 48 h p.i. in C57BL/6 mice, (B) S12023 sseB at 48 h p.i. in gp91phox −/− mice. (TIF) [file ppat.1003070.s009.tif]
