# Supplementary material for: Attenuated Salmonella Typhimurium Lacking the Pathogenicity Island-2 Type 3 Secretion System Grow to High Bacterial Numbers inside Phagocytes in Mice
Source: PLoS Pathog. 2012 Dec 6;8(12):e1003070. doi: 10.1371/journal.ppat.1003070 (PMC3516571; doi:10.1371/journal.ppat.1003070)
Supplement: Protocol S1 — Details of S. enterica mutant generation, model constructions and statistical analyses. (DOCX) [file ppat.1003070.s010.docx]

Protocol S1. Details of *S. enterica* mutant generation, model constructions and statistical analyses

Generation of *S. enterica* mutants

*Generation of S12023* aroA *and SL5559* aroA *mutants*

PCR was used to amplify the kanamycin resistance cassette from pACYC177 [*S1*] with 5’ and 3’ 60 bp homology arms complementary to the flanking regions of *aroA* using the primers ajg668 and ajg669 (Table S3). The correct genomic rearrangements in the resultant mutants were confirmed by PCR and sequencing (data not shown) using the primers ajg656 and ajg657 (Table S3).

*Generation of S12023* sseB aroA *mutant*

PCR was used to amplify the chlormaphenicol resistance cassette from pACYC184 [*S1*] with 5’ and 3’ 60 bp homology arms complementary to the flanking regions of *aroA* using the primers ajg654 and ajg655 (Table S3). The correct genomic rearrangements in the resultant mutants were confirmed by PCR and sequencing (data not shown) using the primers ajg656 and ajg657 (Table S3).

*Generation of S12023* purA *mutant*

PCR was used to amplify the kanamycin resistance cassette from pACYC177 [*S1*] with 5’ and 3’ 60 bp homology arms complementary to the flanking regions of *purA* using the primers ajg688 and ajg689 (Table S3). The correct genomic rearrangements in the resultant mutants were confirmed by PCR and sequencing (data not shown) using the primers and ajg690 and ajg691 (Table S3).

*Generation of S12023* sseB purA *mutant*

PCR was used to amplify the chloramphenicol resistance cassette from pACYC184 [*S1*] with 5’ and 3’ 60 bp homology arms complementary to the flanking regions of *purA* using the primers ajg694 and ajg695 (Table S3). The correct genomic rearrangements in the resultant mutants were confirmed by PCR and sequencing (data not shown) using the primers and ajg690 and ajg691 (Table S3).

*Generation of SL5559* sseB *and SL5560* sseB *mutants*

PCR was used to amplify the chloramphenicol resistance cassette from pACYC184 [*S1*] with a 5’ 60 bp homology arm complementary to the flanking region of *sseB* and a 3’ 60 bp homology arm complementary to the last 15 bases of *sseB* and the flanking region of *sseB* using the primers ajg670 and ajg671 (Table S3). The correct genomic rearrangements in the resultant mutants were confirmed by PCR and sequencing (data not shown) using the primers ajg672 and ajg673 (Table S3).

*Generation of SL1344* ssaV *and C5* ssaV *mutants*

PCR was used to amplify the chloramphenicol resistance cassette from pACYC184 [*S1*] with 5’ and 3’ 60 bp homology arms complementary to the flanking regions of *ssaV* using the primers ssaV F and ssaV R. The correct genomic rearrangements in the resultant mutants were confirmed by PCR (data not shown) using the primers ssaVF Test and ssaVR Test (Table S3). Additionally, mutants were verified by Southern hybridization using a probe generated by PCR, and designed to anneal upstream of the mutated gene of interest (data not shown). The probe upstream of *ssaV* was amplified using primers ssaV probe F and ssaV probe R (Table S3).

*Generation of S12023* spiC *mutant*

PCR was used to amplify the chlormaphenicol resistance cassette from pACYC184 [*S1*] with 5’ and 3’ 60 bp homology arms complementary to the flanking regions of *spiC* using the primers ajg730 and ajg731 (Table S3). The correct genomic rearrangements in the resultant mutants were confirmed by PCR and sequencing (data not shown) using the primers ajg696 and ajg699 (Table S3).

*Generation of S12023* ssaM *mutant*

PCR was used to amplify the chlormaphenicol resistance cassette from pACYC184 [*S1*] with 5’ and 3’ 60 bp homology arms complementary to the flanking regions of *ssaM*, to delete codons encoding amino acids 10-103 of SsaM [*S2*], using primers ajg791 and ajg792 (Table S3). The correct genomic rearrangements in the resultant mutants were confirmed by PCR and sequencing (data not shown) using the primers ajg767 and ajg768 (Table S3).

*Generation of S12023* spvB and *S12023* sseL spvB *mutants*

PCR was used to amplify the chlormaphenicol resistance cassette from pACYC184 [*S1*] with 5’ and 3’ 60 bp homology arms complementary to the flanking regions of *spvB* using the primers ajg797 and ajg798 (Table S3). The correct genomic rearrangements in the resultant mutants were confirmed by PCR and sequencing (data not shown) using the primers ajg801 and ajg802 (Table S3).

Modeling the intracellular bacterial counts

Due to difficulties in accurately quantifying the exact number of bacteria per cell at very high intracellular bacterial loads, we decided to model the data by grouping the counts into a series of categories. The differences in distributions can then be examined by considering proportions of phagocytes in each category (based on the number of bacteria per cell). Bacterial loads were classified into 5 groups (1, 2, 3-5, 6-10 and >11) and ordinal regression was used to explore the relationships between bacterial load and the variables of interest.

Therefore, if there are *n* infected cells then each cell can be classified into one of five categories as specified above. Letting be a category indicator for cell *i* , then

,

where corresponds to the set of probabilities that cell *i* is in any given category. Therefore for . In order to account for the ordering we model the cumulative probabilities, , as:

where is a linear regression term and are *q* variables of interest. The parameters correspond to a set of latent continuous “cut-points”, such that . Since we include an intercept, then for identifiability we set . The probabilities of category membership are then given as:

This is a so-called proportional odds model (see *e.g.* [3] for more details).

The explanatory variables are dummy variables corresponding to whether an individual *i* belongs to a particular group. For example, if we have measurements from two time points, 0.5 h p.i. and 72 h p.i. say, then this could be represented by a variable , such that

and likewise for other variables. Multiple dummy variables can be employed to account for variables that may contain multiple levels, such as strain [*e.g.* S12023, S12023 *sseB* and S12023 *sseB*(psseB)].

The proportional odds model assumes that these odds ratios do not depend on the cut-points, and hence the ORs can be interpreted as *overall* relative measures of intracellular bacterial loads.

In addition the data consist of multiple repeated measures from different animals, and so it was also felt necessary to account for this in the model specification. We do this by allowing the intercept term to vary by each mouse according to a probability distribution (a so-called random intercepts model). In a Bayesian framework all parameters are treated as random variables in any case, and so we deem this a hierarchical model. Hence

,

and the () allow the intercept to vary according to each of the mice, and is an indicator of which mouse the *i*^th^ cell comes from.

To complete the Bayesian specification, for all models we use independent prior distributions for the parameters, and truncated normal prior distributions for the cut-points, such that

For identifiability we use priors for the parameters, where the standard deviation follows a flat hyperprior over the positive real line.

In order to fit the Bayesian model we use a Metropolis-Hastings Markov chain Monte Carlo framework (see *e.g.* [4,5]), using an adaptive proposal distribution of the form described [6] for joint block updates of and , and independent proposal distributions for component-wise updates of and (where is a fixed constant).

A further advantage with the Bayesian framework is that the marginal posterior for any given parameter is trivial to obtain from the MCMC runs, and corresponds to integrating across the range of the other parameters. Therefore the uncertainty in any given marginal distribution accounts for the uncertainty present in the other parameters of the model. We can then generate 95% credible intervals for any given parameter or combinations of parameters. Unless specified below and in Table S1 we used a proposal jump of , and ran two chains of 100,000 iterations, discarding the first 20,000 as burn-in and thinning the rest to return 1,000 samples. Therefore all estimates presented in this manuscript are generated from 2,000 posterior samples. The output in Figure S5 was generated from 2000 thinned samples from 1,000,000 iterations with the first 500,000 discarded as burn-in.

Modeling the numbers of infected cells per field of view

In this case it was possible to count the number of infected cells per field-of-view with much more accuracy, and so we model the counts directly. Some exploratory analysis (assuming that the data were Poisson distributed) suggested that there was over-dispersion present, and hence we adopted a negative binomial regression model to account for this.

Therefore, if represents the number of infected cells in the *i*^th^ field-of-view , then

,

where

,

with ,

and for all *m* for all *i*. Here *q>m*, and we set the first *m* variables as main effect indicators for each strain, and the remaining *q-m* variables are the main effect for time and the time strains interaction effects. Essentially we allow the shape parameter *r* to vary between strains and the scale parameter to vary between strains and time. As in the previous model we use the random intercepts to account for between-mouse heterogeneity. The prior distributions for , and are as before and we use a prior distribution for each (with mean and variance 1 and 100 respectively). Again, we used an adaptive proposal of the form described in [6] for joint block updates of and , and independent proposal distributions for updates of (where is a fixed constant).

We used a proposal jump of , and ran two chains of 100,000 iterations, discarding the first 20,000 as burn-in and thinning the rest to return 1,000 samples. Therefore all estimates are generated from 2,000 posterior samples. Differences between groups can be explored by comparing the mean counts (equal to for any given set of explanatory variables ) and credible intervals can be interpreted relative to zero (*i.e.* no difference between the means).

Interpreting the odds ratios

In any given bacterial load category (1, 2, 3-5, 6-10 and >11), we can examine differences between groups (*e.g.* strain or time post infection) in terms of the proportional odds ratios (ORs) of belonging to a higher bacterial load category. For example, the first line of Table S1 says that a cell infected with S12023 *sseB* in the liver at the 0.5 h p.i. time point, is 1.1 times more likely to have a higher bacterial load than a cell infected with S12023 wild-type under the same conditions (with 95% credible interval of 0.57–2.0). These ORs can be interpreted relative to a value of 1 (in which the odds of cells having a higher bacterial load is the same for the comparative groups), so in this case the credible interval spans one, and hence there is not strong evidence that the two groups show different odds of higher bacterial loads relative to each other. However, the second comparison S12023 *sseB* vs. S12023 wild-type in the liver at the 72 h p.i. time point (the second line of Table S1), provides much stronger evidence that the bacterial loads are higher in the mutant S12023 *sseB* than in the wild-type strain [OR: 4.2, 95% CI: (2.7,6.3)].

**References**

1. Chang CY, Cohen SN (1978) Construction and characterization of amplifiable multicopy DNA cloning vehicles derived from the P15A cryptic miniplasmid. J Bacteriol 134: 1141-1156.
2. Yu X-J, Liu M, Holden DW (2004) SsaM and SpiC interact and regulate secretion of *Salmonella* pathogenicity island 2 type III secretion system effectors and translocators. Mol Microbiol 54: 604-619.
3. Congdon P (2005) Bayesian Models for Categorical Data, Wiley.
4. Gilks WR, Richardson S, Spiegelhalter DF (1996) Markov Chain Monte Carlo In Practice. Publisher, Chapman and Hall.
5. Gamerman D, Lopes HF (2006) Markov Chain Monte Carlo: Stochastic Simulation for Bayesian Inference. 2^nd^ Edition. Publisher, CRC Press.
6. Roberts GO, Rosenthal JS (2009) Examples of Adaptive MCMC. J. Comput Grap Stat 18: 349-367.
