# Supplementary material for: Attenuated Salmonella Typhimurium Lacking the Pathogenicity Island-2 Type 3 Secretion System Grow to High Bacterial Numbers inside Phagocytes in Mice
Source: PLoS Pathog. 2012 Dec 6;8(12):e1003070. doi: 10.1371/journal.ppat.1003070 (PMC3516571; doi:10.1371/journal.ppat.1003070)
Supplement: Table S1 — Posterior means and 95% credible intervals for proportional odds ratios from hierarchical Bayesian ordinal regression. (DOCX) [file ppat.1003070.s011.docx]

**Table S1.** **Posterior means and 95% credible intervals for proportional odds ratios from hierarchical Bayesian ordinal regression**

|  |  |  |  | **Credible interval** | |
| --- | --- | --- | --- | --- | --- |
| **Odds ratio** | **Organ** | **Time** | **Mean** | **2.5%** | **97.5%** |
| ^a^S12023 *sseB* - S12023 | Liver | 0.5 | 1.1 | 0.57 | 2.0 |
|  |  | 72 | 4.2 | 2.7 | 6.3 |
|  | Spleen | 0.5 | 1.1 | 0.48 | 2.3 |
|  |  | 72 | 3.8 | 2.5 | 5.7 |
| ^a^S12023 *sseB*(psseB) - S12023 | Liver | 0.5 | 1.3 | 0.65 | 2.2 |
|  |  | 72 | 0.75 | 0.48 | 1.1 |
|  | Spleen | 0.5 | 1.4 | 0.61 | 2.8 |
|  |  | 72 | 0.54 | 0.35 | 0.82 |
| ^a^S12023 *sseB* - S12023 *sseB*(psseB) | Liver | 0.5 | 0.93 | 0.48 | 1.7 |
|  |  | 72 | 5.7 | 3.6 | 8.5 |
|  | Spleen | 0.5 | 0.86 | 0.38 | 1.7 |
|  |  | 72 | 7.2 | 4.6 | 11 |
| ^a^S12023 *sseB* (6 h p.i.) – S12023 *sseB* (0.5 h p.i.) | Liver |  | 4.8 | 2.1 | 9.6 |
|  | Spleen |  | 6.6 | 2.2 | 18 |
| ^a^S12023 *sseB* (24 h p.i.) – S12023 *sseB* (0.5 h p.i.) | Liver |  | 15 | 6.8 | 31 |
|  | Spleen |  | 24 | 8.5 | 60 |
| ^a^S12023 *sseB* (48 h p.i.) – S12023 *sseB* (0.5 h p.i.) | Liver |  | 20 | 8.9 | 40 |
|  | Spleen |  | 60 | 21 | 151 |
| ^a^S12023 *sseB* (72 h p.i.) – S12023 *sseB* (0.5 h p.i.) | Liver |  | 20 | 8.8 | 41 |
|  | Spleen |  | 85 | 29 | 211 |
| ^b^S12023 *aroA* - S12023 *sseB* | Liver | 0.5 | 1.1 | 0.40 | 2.3 |
|  | Spleen | 0.5 | 1.7 | 0.52 | 4.1 |
|  | Liver | 72 | 0.12 | 0.06 | 0.23 |
|  | Spleen | 72 | 0.15 | 0.08 | 0.27 |
| ^b^S12023 *purA* - S12023 *sseB* | Liver | 0.5 | 1.0 | 0.42 | 2.1 |
|  | Spleen | 0.5 | 1.3 | 0.40 | 3.3 |
|  | Liver | 72 | 0.18 | 0.08 | 0.32 |
|  | Spleen | 72 | 0.13 | 0.07 | 0.25 |
| ^b^S12023 *sseB aroA* - S12023 *sseB* | Liver | 0.5 | 1.4 | 0.57 | 2.7 |
|  | Spleen | 0.5 | 3.0 | 1.1 | 7.1 |
|  | Liver | 72 | 0.94 | 0.48 | 1.7 |
|  | Spleen | 72 | 0.76 | 0.38 | 1.3 |
| ^b^S12023 *sseB purA* - S12023 *sseB* | Liver | 0.5 | 1.4 | 0.56 | 2.7 |
|  | Spleen | 0.5 | 2.5 | 0.90 | 6.2 |
|  | Liver | 72 | 0.62 | 0.31 | 1.1 |
|  | Spleen | 72 | 0.28 | 0.14 | 0.48 |
| ^b^S12023 *purA* - S12023 *aroA* | Liver | 0.5 | 1.1 | 0.44 | 2.3 |
|  | Spleen | 0.5 | 0.90 | 0.29 | 2.1 |
|  | Liver | 72 | 1.5 | 0.70 | 2.7 |
|  | Spleen | 72 | 0.94 | 0.45 | 1.7 |
| ^b^S12023 *sseB aroA* - S12023 *aroA* | Liver | 0.5 | 1.4 | 0.62 | 3.1 |
|  | Spleen | 0.5 | 2.0 | 0.82 | 4.6 |
|  | Liver | 72 | 8.0 | 3.9 | 15 |
|  | Spleen | 72 | 5.4 | 2.6 | 10 |
| ^b^S12023 *sseB purA* - S12023 *aroA* | Liver | 0.5 | 1.4 | 0.59 | 3.0 |
|  | Spleen | 0.5 | 1.72 | 0.63 | 4.0 |
|  | Liver | 72 | 5.3 | 2.7 | 9.4 |
|  | Spleen | 72 | 1.9 | 0.99 | 3.5 |
| ^b^S12023 *sseB aroA* - S12023 *purA* | Liver | 0.5 | 1.4 | 0.63 | 2.9 |
|  | Spleen | 0.5 | 2.6 | 0.90 | 6.3 |
|  | Liver | 72 | 5.7 | 2.9 | 10 |
|  | Spleen | 72 | 6.1 | 2.9 | 11 |
| ^b^S12023 *sseB purA* - S12023 *purA* | Liver | 0.5 | 1.4 | 0.60 | 2.9 |
|  | Spleen | 0.5 | 2.2 | 0.72 | 5.1 |
|  | Liver | 72 | 3.7 | 1.9 | 6.7 |
|  | Spleen | 72 | 2.2 | 1.1 | 3.9 |
| ^b^S12023 *sseB* *purA* - *sseB* *aroA* | Liver | 0.5 | 1.1 | 0.47 | 2.2 |
|  | Spleen | 0.5 | 0.90 | 0.37 | 1.9 |
|  | Liver | 72 | 0.69 | 0.34 | 1.2 |
|  | Spleen | 72 | 0.38 | 0.19 | 0.71 |
| ^a^S12023 sseB in gp91-/- phox mice – S12023 sseB in C57BL/6 mice | Liver | 48 | 0.16 | 0.06 | 0.34 |
|  | Spleen | 48 | 0.40 | 0.15 | 0.83 |

^a^We used a proposal jump of , and ran two chains of 100,000 iterations, discarding the first 20,000 as burn-in and thinning the rest to return 1,000 samples.

^b^The output was generated from 2,000 thinned samples from 1,000,000 iterations with the first 500,000 discarded as burn-in.
