# Supplementary material for: Attenuated Salmonella Typhimurium Lacking the Pathogenicity Island-2 Type 3 Secretion System Grow to High Bacterial Numbers inside Phagocytes in Mice
Source: PLoS Pathog. 2012 Dec 6;8(12):e1003070. doi: 10.1371/journal.ppat.1003070 (PMC3516571; doi:10.1371/journal.ppat.1003070)
Supplement: Table S2 — Differences between mean number of infected cells per field-of-view, from hierarchical Bayesian negative binomial regression. (DOCX) [file ppat.1003070.s012.docx]

**Table S2. Differences between mean number of infected cells per field-of-view, from hierarchical Bayesian negative binomial regression**

|  |  | **Credible interval** | |
| --- | --- | --- | --- |
| **Comparison** | **Mean difference** | **2.5%** | **97.5%** |
| S12023 *sseB* (72 h p.i.) - S12023 (72 h p.i.) | -28.2 | -40.9 | -17.6 |
| S12023 *sseB*(psseB) (72 h p.i.) - S12023 (72 h p.i.) | -10.5 | -25.6 | 2.8 |
| S12023 *sseB* (72 h p.i.) - S12023 *sseB*(psseB) (72 h p.i.) | -17.7 | -26.0 | -11.1 |
| S12023 *sseB* (0.5 h p.i.) - S12023 *sseB* (72 h p.i.) | 0.3 | -1.4 | 2.4 |
| S12023 *spiC* (0.5 h p.i.) - S12023 *spiC* (72 h p.i.) | -0.1 | -1.7 | 1.4 |
| S12023 *aroA* (0.5 h p.i.) - S12023 *aroA* (72 h p.i.) | -2.4 | -4.8 | -0.6 |
| S12023 *sseB aroA* (0.5 h p.i.) - S12023 *sseB aroA* (72 h p.i.) | 1.9 | 0.2 | 3.0 |
| S12023 *purA* (0.5 h p.i.) - S12023 *purA* (72 h p.i.) | -8.6 | -21.5 | 1.6 |
| S12023 *sseB purA* (0.5 h p.i.) - S12023 *sseB purA* (72 h p.i.) | 2.7 | -0.7 | 6.5 |
| S12023 *sseB* in *gp91*^-/-^ *phox* mice (48 h p.i.) - S12023 *sseB* in C57BL/6 mice (48 h p.i.) | 16.02 | 5.91 | 30.30 |
