# Supplementary material for: Attenuated Salmonella Typhimurium Lacking the Pathogenicity Island-2 Type 3 Secretion System Grow to High Bacterial Numbers inside Phagocytes in Mice
Source: PLoS Pathog. 2012 Dec 6;8(12):e1003070. doi: 10.1371/journal.ppat.1003070 (PMC3516571; doi:10.1371/journal.ppat.1003070)
Supplement: Table S3 — Primer sequences used in this study. (DOCX) [file ppat.1003070.s013.docx]

**Table S3. Primer sequences used in this study.**

| **Primer** | **Sequence 5’ to 3’** |
| --- | --- |
| ajg654 | TTTTCATCCCCACGGCCAGTCTGTGGGGTTTTTATTTCTGTTTTTTGAGAGTTGAGTTTCCGACGCACTTTGCGCCGAAT |
| ajg655 | TCACCGTCCCGCCCCGCATTCAGAAGACAACGCGGTCAGCTGCCCGACCGCGCGGCTCAGGCATAAGCGTGATAAAAATA |
| ajg656 | CGCCATGCCGATTGAAGGGGT |
| ajg657 | TAATCTTCCGTTGATAACTAT |
| ajg668 | TTTTCATCCCCACGGCCAGTCTGTGGGGTTTTTATTTCTGTTTTTTGAGAGTTGAGTTTCCTCAAAATCTCTGATGTTACATTGC |
| ajg669 | ATAAAAATAGTGCGAATACGGACTCGGCGCGCCAGCCCGTCGACTGGCGCAACAGAAGACTTAGAAAAACTCATCGAGCATC |
| ajg670 | CAACTTAATGCTCAAGCCCCGGTGGAGATACCGTCAGGAAAAACAAAAAGGTAAAGCATACGACGCACTTTGCGCCGAAT |
| ajg671 | AAAACCGCATCGTGTCATGTGCCTGTTGTAGGGTCGGGTCTTTTTTCATGAGTACGTTTTTTACGCCCCGCCCTGCCACT |
| ajg672 | TTGCCGGGGTTAACCAGGAGG |
| ajg673 | CGTGGCATAGCGATACAGAGT |
| ajg688 | GCTGTATATCTGAAAAAGCGATGGTAGAATCCATTTTTAAGCAAACGGTGATTTTGAAAACTCAAAATCTCTGATGTTACATTGC |
| ajg689 | ACACAGCAATGTATCACACTGTTAGCCCGGCAAGCAAAATATCTGCCAGGCGTACCAGATTTAGAAAAACTCATCGAGCATC |
| ajg690 | TTGTGGTCTACTACATGTTGA |
| ajg691 | GTGTCGCTAATTTATTTCAGT |
| ajg694 | GCTGTATATCTGAAAAAGCGATGGTAGAATCCATTTTTAAGCAAACGGTGATTTTGAAAACGACGCACTTTGCGCCGAAT |
| ajg695 | TCACCGTCCCGCCCCGCATTTAGACCATGCGGACCGTCTATAAAACGAACGGCCCGATTGTCACACTATGTAACGACACA |
| ajg696 | GGCGATGTAAAAACATCGTAA |
| ajg699 | CCAGGCGGAATTTTTCCACTA |
| ajg730 | AATGTGAATCAGGCTTTTTACGGATGTGGTTGTGAGCGAATTTGATAGAAACTCCCATTTCGACGCACTTTGCGCCGAAT |
| ajg731 | CTCTTTGCTGTATTGAGTATAAATAGTAAAATTAAGATTAAACGTTTATTTACTACCATTTTACGAAAAGCCCTGCCACT |
| ajg767 | AGATAAATCAGGTTTTATTCT |
| ajg768 | GATTAATAACACCGTCGCCAG |
| ajg791 | AATTGGCTTTCTGGCTCATCATGAGGCGTCAGGATGGATTGGGATCTCATTACTGAACGTCGACGCACTTTGCGCCGAAT |
| ajg792 | CTAACCATGAACGCATTGCGACTCCAGAAATTTTATTTGTCGATGATGTAATCGTAACCATTACGCCCCGCCCTGCCACT |
| ajg797 | CACTTTTGAACAGGCCGTAGAGCAGACGCTGTAAGCAGAAAATATACCTGGCCATCGTCAGACGGCCAGTTTCAGGAGATAGTGTCGACGCACTTTGCGCCGAAT |
| ajg798 | GGTGACGGAAAAATCTGGGGTTCCGGAGTATAACCCCTTATAGAGCTAGGCCGCTCATACCACTTCTGGAATAGATTCTTAGTATTTACGCCCCGCCCTGCCACT |
| ajg801 | AGAATCTGAGCGACGTCATTG |
| ajg802 | TAAAGAAGGGTAATGGCAAAA |
| ssaV F | CAGCTCCGCCGAGCTCTGGTTACGATTACATCATCGACAAATAAAATTTCTGGAGTCGCATGAGACGTTGATCGGCACGT |
| ssaV R | CGGCCCCATCGACAATAACCATCGGGGGGCGGATATTTCAGCCTCAGACGTTGCATCAATGCTTATTATCACTTATTCAGGCG |
| ssaVF Test | TATGTTCTGGCGGCAAGG |
| ssaVR Test | CTCCAGGCTTTATACAGCACA |
| ssaV probe F | CTTATCGCAGTGGTTCAGAC |
| ssaV probe R | TTGAAAGCCAGGTATCAGAA |
